# Transcriptional activation of auxin biosynthesis drives developmental reprogramming of differentiated cells

**DOI:** 10.1093/plcell/koac218

**Published:** 2022-08-04

**Authors:** Yuki Sakamoto, Ayako Kawamura, Takamasa Suzuki, Shoji Segami, Masayoshi Maeshima, Stefanie Polyn, Lieven De Veylder, Keiko Sugimoto

**Affiliations:** Department of Biological Sciences, Graduate School of Science, The University of Tokyo, Tokyo 113-0033, Japan; Center for Sustainable Resource Science, RIKEN, Yokohama 230-0045, Japan; Center for Sustainable Resource Science, RIKEN, Yokohama 230-0045, Japan; Department of Biological Chemistry, College of Bioscience and Biotechnology, Chubu University, Kasugai 487-8501, Japan; Division of Evolutionary Biology, National Institute for Basic Biology, Okazaki 444-8585, Japan; Department of Basic Biology, School of Life Science, The Graduate University for Advanced Studies, SOKENDAI, Okazaki 444-8585, Japan; Department of Biological Chemistry, College of Bioscience and Biotechnology, Chubu University, Kasugai 487-8501, Japan; Department of Plant Biotechnology and Bioinformatics, Ghent University, Ghent B-9052, Belgium; VIB Center for Plant Systems Biology, Ghent B-9052, Belgium; Department of Plant Biotechnology and Bioinformatics, Ghent University, Ghent B-9052, Belgium; VIB Center for Plant Systems Biology, Ghent B-9052, Belgium; Department of Biological Sciences, Graduate School of Science, The University of Tokyo, Tokyo 113-0033, Japan; Center for Sustainable Resource Science, RIKEN, Yokohama 230-0045, Japan

## Abstract

Plant cells exhibit remarkable plasticity of their differentiation states, enabling regeneration of whole plants from differentiated somatic cells. How they revert cell fate and express pluripotency, however, remains unclear. In this study, we demonstrate that transcriptional activation of auxin biosynthesis is crucial for reprogramming differentiated Arabidopsis (*Arabidopsis thaliana*) leaf cells. Our data show that interfering with the activity of histone acetyltransferases dramatically reduces callus formation from leaf mesophyll protoplasts. Histone acetylation permits transcriptional activation of *PLETHORA*s, leading to the induction of their downstream *YUCCA1* gene encoding an enzyme for auxin biosynthesis. Auxin biosynthesis is in turn required to accomplish initial cell division through the activation of G2/M phase genes mediated by MYB DOMAIN PROTEIN 3-RELATED (MYB3Rs). We further show that the AUXIN RESPONSE FACTOR 7 (ARF7)/ARF19 and INDOLE-3-ACETIC ACID INDUCIBLE 3 (IAA3)/IAA18-mediated auxin signaling pathway is responsible for cell cycle reactivation by transcriptionally upregulating *MYB3R4*. These findings provide a mechanistic model of how differentiated plant cells revert their fate and reinitiate the cell cycle to become pluripotent.

## Teaser

Reprogramming of mature plant cells requires histone acetylation-mediated activation of auxin biosynthesis to reinitiate the mitotic cell cycle.

## Introduction

Tight coordination of cell proliferation and differentiation is central to optimizing plant organ development. Typically, during a normal developmental program, meristematic cells continue to proliferate until they start to differentiate, and once differentiated, the somatic cells usually remain mitotically inactive (Beemster et al., 2005; Dello Ioio et al., 2008; Andriankaja et al., 2012). Such a relationship between mitosis and cellular differentiation status should also be critical during developmental reprogramming through which plant somatic cells convert their fates and regenerate new tissues or organs. Previous studies indeed suggest that reactivation of somatic cell division contributes to cell fate conversion and acquisition of pluripotency, presumably by helping cells to dilute existing identity and activate a new developmental program (Christianson and Warnick, 1983; Zhang et al., 2017; Ikeuchi et al., 2019). Uncovering how differentiated cells reinitiate cell division is, therefore, crucial to understand how somatic cells initiate reprogramming.

Mesophyll cells in mature leaves have fully developed organelles such as chloroplasts, and they usually do not divide in intact plant tissues. When they are isolated by cell wall digestion as single cells called protoplasts and cultivated under phytohormone-containing conditions, however, they reinitiate the cell cycle, grow into an unorganized cell mass called callus, and even regenerate whole plants (Takebe et al., 1971). It has thus been clearly demonstrated that differentiated leaf cells can change their fate and take on a meristematic, pluripotent state. Previous studies have described the cytological and physiological properties of protoplasts during cell cycle reinitiation in several plant species, including tobacco (*Nicotiana tabacum*) and Arabidopsis (*Arabidopsis thaliana*). Protoplasts, for instance, undergo cell wall reconstruction (Kao et al., 1970; Schilde-Rentschler, 1977) and changes in the structure and subcellular localization of organelles (Thomas and Rose, 1983; Sheahan et al., 2007). At the physiological level, cell cycle reinitiation is known to occur with the neutralization of reactive oxygen species) (De Marco and Roubelakis-Angelakis, 1996; Tiew et al., 2015) and phytohormone production (Pasternak et al., 2002). Additionally, several studies have demonstrated that freshly isolated protoplasts possess a more open chromatin state compared to intact leaf cells, possibly caused by drastic changes in epigenetic modifications (Zhao et al., 2001; Williams et al., 2003). Consistent with this, Chupeau et al. (2013) reported that Arabidopsis protoplasts undergo dynamic transcriptomic reprogramming during early phases of incubation. Despite these reported findings, the molecular mechanisms that drive cell cycle reinitiation from differentiated cells remain obscure, partly due to a lack of experimental systems to quantitatively assess this cellular process. To overcome this problem, we improved the culture system for Arabidopsis leaf mesophyll protoplasts and then performed quantitative genetic, physiological, and cell biological analyses. Using these approaches, we demonstrate that transcriptional activation of auxin biosynthesis is essential to promote cell cycle reactivation in differentiated cells.

## Results

### Arabidopsis leaf mesophyll protoplasts reprogram and regenerate shoots in vitro

**Figure 1:**
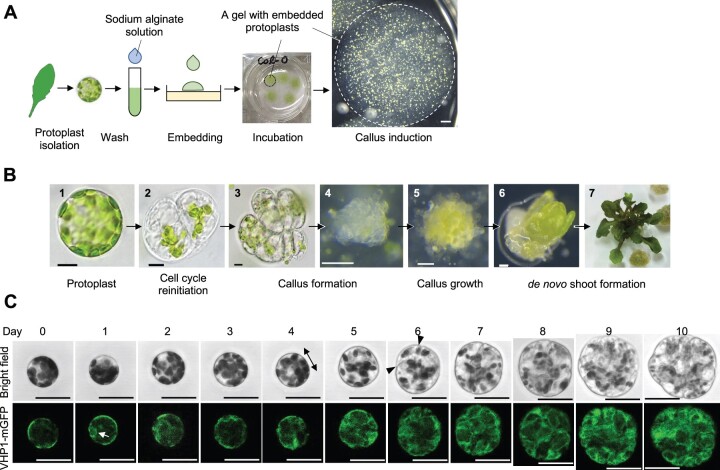
Leaf mesophyll protoplasts reprogram into a pluripotent state and regenerate shoots in vitro. A, Diagram showing the key steps for protoplast isolation and callus induction. The circles with dashed lines indicate a sodium alginate gel that contains protoplasts. B, Light microscopy images of a freshly isolated leaf mesophyll protoplast (1), a protoplast that has undergone the first cell division (2), callus formed from a protoplast (3–5), and new shoots formed from protoplast-derived callus (6 and 7). C, Time-lapse confocal microscopy images of a leaf mesophyll protoplast undergoing cellular reprograming from Days 0 to 10. Vacuolar morphology is visualized by VHP1-mGFP. The double-headed arrow indicates the direction of cell elongation and arrowheads mark the plane of initial cell division. The white arrow highlights the initial appearance of vacuolar strand-like structures. Scale bars are 10 μm (1–3 in A), 100 μm (4–6 in A), 1 mm (B), and 30 μm (C).

We first established a new experimental pipeline to reproducibly induce callus formation from Arabidopsis leaf protoplasts (Figure 1A). Protoplasts with high viability (98.66 ± 0.19%) were isolated from mature rosette leaves, embedded into sodium alginate gels, and cultured in protoplast callus induction medium (PCIM) supplemented with 2,4-dichlorophenoxyacetic acid (2,4-D) and thidiazuron (TDZ) as auxin and cytokinin, respectively (Figure 1A; Supplemental Figure S1A; Supplemental Table S1). After a 14-day incubation, around 2% of the total embedded protoplasts developed into callus, enabling quantitative evaluation of this phenotype (Supplemental Figure S1B). When these protoplast-derived calli were transferred to callus growth medium (CGM) and subsequently to shoot induction medium (SIM), they continued to proliferate and regenerate shoots (Figure 1B), indicating that these protoplasts acquire competence to form shoot meristems.

To uncover the cellular basis for developmental reprogramming, we first determined the original identity of dividing protoplasts. The *pCAB3:H2A-eGFP* reporter, which drives nuclear accumulation of HISTONE 2A-6 (H2A) fused with enhanced green fluorescent protein (eGFP) under the control of a promoter sequence of a mesophyll cell-specific gene *CHLOROPHYLL A/B BINDING PROTEIN 3* (*CAB3*) (Susek et al., 1993), was expressed in most freshly isolated protoplasts (Supplemental Figure S1C). Using a time-lapse live imaging system that tracks reprogramming of individual protoplasts over time (Supplemental Figure S1D), we confirmed that 98.78% (162 out of 164) of protoplasts that underwent cell division displayed mesophyll cell-like characteristics at Day 0 based on H2A-eGFP expression and/or the appearance and density of chloroplasts (Pandey et al., 2002) (Supplemental Figure S1, E–G). The two remaining H2A-eGFP-negative protoplasts also contained chloroplasts, but they resembled guard cells based on their lower chloroplast density than that in mesophyll cells (Pandey et al., 2002) (Supplemental Figure S1H). These observations establish that most protoplasts in our experimental setup initially have mesophyll cell identities.

Closer examination of early morphological changes via time-lapse imaging revealed that protoplasts underwent their first cell division after Day 4 and, consistent with Chupeau et al. (2013), many divided at Day 6 or 7 (Figure 1C; Supplemental Figure S1I). As described previously (Kao et al., 1970; Schilde-Rentschler, 1977), most of them elongated for 1–3 days before initial cell division (Supplemental Figure S1, J and 1K). We have previously shown that vacuolar morphology, visualized by monomeric GFP (mGFP)-tagged VACUOLAR H^+^-PYROPHOSPHATASE 1 (VHP1), changes dynamically as cells transit from proliferative to differentiated phases (Segami et al., 2014). Time-lapse imaging of protoplasts carrying *pVHP1:VHP1-mGFP* showed that freshly isolated protoplasts had a single large vacuole occupying most of the cell volume (Sheahan et al., 2007) (Day 0 in Figure 1C). Strand-like structures, however, started to appear inside the vacuole either before or when the cells started elongating, and vacuoles underwent extensive compartmentalization as cells progressed through successive divisions (Figure 1C).

In parallel with the reactivation of cell proliferation, cellular dedifferentiation, that is the loss of existing traits, is another important aspect of developmental reprogramming. It was previously reported that during in vitro transdifferentiation of mesophyll cells into xylem cells, the expression levels of genes that characterize mesophyll cells, for example, photosynthetic genes such as *CAB3* and *RIBULOSE BISPHOSPHATE CARBOXYLASE SMALL CHAIN 1A*, are immediately decreased, reflecting the loss of mesophyll identity (Kondo et al., 2015). To investigate when protoplasts start to dedifferentiate, we examined the expression of genes involved in chloroplast functions in mature leaves and protoplasts at early incubation steps by RNA sequencing (Supplemental Figure S2; Supplemental Data Set 1). Since leaf samples contained other cell types such as epidermis and vascular cells that do not contain chloroplasts, we cannot make quantitative assessments between these datasets. It was, however, clear that many genes encoding photosynthetic components, including subunits of light-harvesting complexes, were downregulated during protoplast isolation and/or following several days of incubation. In contrast, genes involved in chloroplast fission, the step important for chloroplast inheritance during cell division, were upregulated within 2 days of incubation, implying that protoplasts initiated transcription to prepare for cell cycle reinitiation by this time. Importantly, many chloroplast-related genes that become downregulated in cultured protoplasts are known to be significantly upregulated as cells transit from proliferative to differentiated phases in growing Arabidopsis leaves (Andriankaja et al., 2012) (Supplemental Figure S2), implying that the changes in expression of these genes in protoplasts reflect initiation of cellular dedifferentiation. These results thus suggest that cellular dedifferentiation starts as early as during protoplast isolation and the early steps of culture, prior to initial cell division.

We also observed protoplasts that elongated or expanded isotropically without cell division (Supplemental Figure S3, A and B). These protoplasts formed strand-like structures of vacuoles similar to those in dividing protoplasts, but they did not undergo or maintain similar levels of compartmentalization (Supplemental Figure S3, C–E), suggesting that the sustained vacuolar compartmentalization marks protoplasts reprogrammed to divide. In addition, other protoplasts shrank or showed no apparent changes in size or shape (Supplemental Figure S3, A and B). A previous study reported that cell wall regeneration occurs within 2 days after protoplast isolation (Kuki et al., 2017). Consistent with this previous study, our RNA-seq data showed that many cell wall biosynthesis genes, including *CELLULOSE SYNTHASE*s and *CELLULOSE SYNTHASE-LIKE*s, were sharply upregulated by Day 2 (Supplemental Data Set 2). Since many protoplasts started to shrink by Day 2 (Supplemental Figure S3B), cell wall regeneration might be a key step for protoplasts to reacquire the mechanical strength at early steps and advance the subsequent reprogramming processes.

### Histone acetylation is required for cell cycle reinitiation in protoplasts

**Figure 2:**
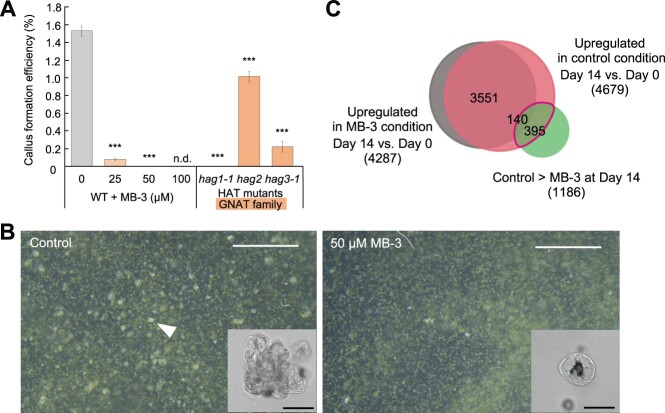
Histone acetylation is required to reinitiate cell division in leaf mesophyll protoplasts. A, Callus formation efficiency of MB3-treated WT, *hag1-1*, *hag2* and *hag3-1* protoplasts at Day 14. Four microliters of DMSO with or without MB-3 were added to PCIM at the indicated final concentration at the beginning of culture (Day 0). Data are represented as mean ± standard error of the mean (sem). *n* = 40 from eight biological replicates for the WT control, 15 from three biological replicates for *hag1-1* and *hag2*, and 20 from four biological replicates for all others. ***P < 0.001 (two-tailed Welch’s *t* test compared to WT control). n.d. not determined. B, Bright field images of WT protoplasts incubated in control (left) and 50-μM MB-3 condition (right) for 14 days. The white arrowhead indicates a single callus. The bottom right insets show confocal microscopy images of callus cells (left) and an undivided protoplast (right). C, A Venn diagram of genes significantly upregulated in control and 50-μM MB-3 conditions. Numbers in brackets show total numbers of genes included in each group. The lower-right circle indicates 1186 genes that show significantly higher expression in the control compared to the MB-3 condition at Day 14. The outline highlights 535 (140 + 395) genes that show significantly stronger upregulation in the control compared to that in the MB-3 condition. Scale bars are 1 mm (B) and 50 μm (insets in B).

To elucidate the molecular mechanisms of developmental reprogramming, we next focused on the epigenetic modifications that protoplasts undergo during the reprogramming process. Williams et al. (2003) reported that levels of histone acetylation, which is thought to promote gene expression (Shahbazian and Grunstein, 2007), increase in freshly isolated protoplasts compared to those in intact leaves. This suggests that histone acetylation ushers in transcriptional changes in protoplasts during early stages of culture, thus driving developmental reprogramming. Given that GCN5-related N-terminal acetyltransferases (GNAT)/MOZ, Ybf2, Sas2 and Tip60 (MYST) family histone acetyltransferases (HATs) regulate the expression of key genes in several regeneration contexts, such as during wound-induced callus formation and in vitro shoot regeneration from explants (Kim et al., 2018; Rymen et al., 2019), we first tested whether an inhibitor of this family of HATs, MB-3, interferes with protoplast reprograming. As shown in Figure 2, A and B, application of MB-3 to wild-type (WT) protoplasts strongly reduced callus formation efficiency at Day 14, with protoplasts largely failing to reinitiate cell division. Applying MB-3 at later time points caused similar defects in callus formation (Supplemental Figure S4A), suggesting that histone acetylation is also required for successive cell divisions. Additionally, an inhibitor of CBP-family HATs, C646, strongly reduced callus formation, whereas garcinol, an inhibitor for p300 and PCAF HATs in humans, had a much milder effect (Supplemental Figure S4B). Although the target specificity of these inhibitors is not well worked out in plants, these data imply that multiple HATs from different subfamilies contribute to protoplast reprogramming. Consistently, we found that among 12 HATs in Arabidopsis, HAT OF THE GNAT/MYST SUPERFAMILY 1 (HAG1), HAG3, and HISTONE ACETYLATION OF THE TAFII250 FAMILY 1 (HAF1) are the key HATs involved in protoplast reprogramming since their single mutants were severely impaired in callus formation (Figure 2A; Supplemental Figure S4, C–E). Interestingly, we did not see any clear association between these single HAT mutant phenotypes and the expression patterns of HAT genes in the control culture condition (Figure 2A; Supplemental Figure S4, C and D and Supplemental Data Set 3). It is thus likely that these HAT activities are regulated at levels other than the transcriptional level.

To investigate how histone acetylation regulates protoplast reprograming through transcriptional regulation, we examined gene expression via RNA-seq in protoplasts cultured with or without 50-μM MB-3. Considering that cell division occurred in only a small percent of protoplasts (Supplemental Figure S3A) and was not synchronized over time (Supplemental Figure S1, I and J), we harvested freshly isolated protoplasts as well as the protoplasts cultured for 14 days, when we expected that the majority of division-competent protoplasts would have undergone the first cell division, to maximize the number of detectable differentially expressed genes (DEGs). We detected more than 7,000 DEGs for both control and MB-3 conditions compared to the freshly isolated protoplasts (Figure 2C, Supplemental Figure S4F and Supplemental Data Set 4). Importantly, the number of downregulated genes was comparable between control and MB-3 conditions (Supplemental Figure S4F). In addition, the expression levels of key metabolic genes, for instance those encoding components of the tricarboxylic acid (TCA) cycle and respiratory chain, which are linked with cellular survival (Martínez-Reyes and Chandel, 2020), were generally similar in the control and MB-3 condition (Supplemental Data Set 5), implying that the cellular physiological status of MB3-treated protoplasts was not severely compromised. Consistently, fluorescein diacetate (FDA) staining showed the comparable viability of the protoplasts cultured in the two conditions (Supplemental Figure S4G), further supporting that MB-3 does not have general toxic effects on the protoplasts.

**Figure 3:**
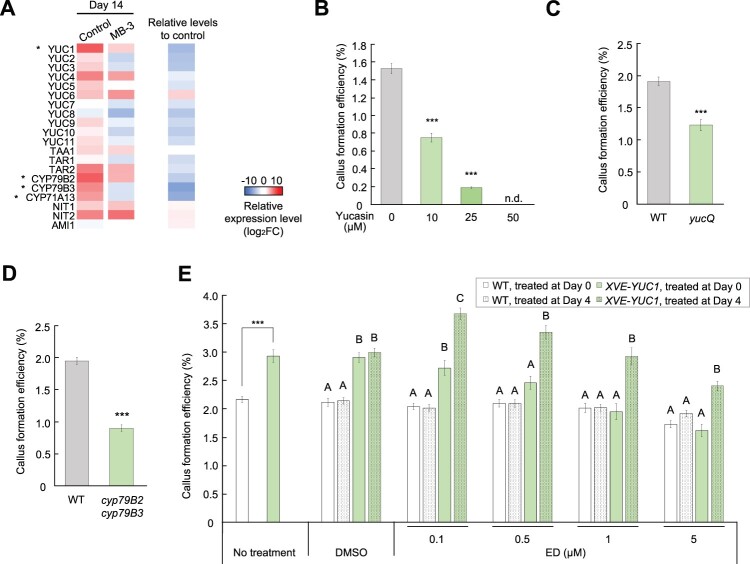
Auxin biosynthesis is required to reinitiate cell division in leaf mesophyll protoplasts. A, Heat map of the transcriptional changes for representative genes implicated in IAA biosynthesis. The left two columns show the expression levels in the control and MB-3 conditions at Day 14 as values normalized (log_2_FC) to Day 0. The “Relative levels to control” column shows the differential expression levels in the MB-3 condition compared to that in the control at Day 14 as relative values (log_2_FC). Asterisks mark the genes that are included from the 535 upregulated genes in Figure 2C. B, Callus formation efficiency of yucasin-treated WT protoplasts. Data are represented as mean ± sem. *n* = 30 from six biological replicates for the WT control and 15 from three biological replicates for the others. ^***^P < 0.001 (two-tailed Welch’s *t* test compared to WT control). n.d. not determined. C, Callus formation efficiency of WT and *yucQ* protoplasts. Data are represented as mean ± sem. *n* = 15 from three biological replicates. ^***^P < 0.001 (two-tailed Student’s *t* test compared to WT). D, Callus formation efficiency of WT and *cyp79B2 cyp79B3* protoplasts. Data are represented as mean ± sem. *n* = 15 from three biological replicates. ^***^P < 0.001 (two-tailed Student’s *t* test compared to WT). E, Callus formation efficiency of *XVE-YUC1* protoplasts. Four microliters of DMSO with or without ED was added to PCIM at the indicated final concentration at Days 0 or 4 of culture. Data are represented as mean ± sem. *n* = 15 from three biological replicates. For “No treatment”, ^***^P < 0.001 (two-tailed Welch’s *t* test compared to WT under the same treatment). For the others, different letters indicate significant differences based on one-way analysis of variance (ANOVA) with post hoc Tukey’s HSD test (*P* < 0.001) within each treatment group.

Since histone acetylation is associated with activation of transcription, we focused on the 535 genes that showed significantly stronger upregulation in the control condition compared to that in the MB-3 condition (Figure 2C; Supplemental Data Set 4). Gene ontology (GO) analysis of the 535 upregulated genes revealed strong fold enrichment of genes implicated in biosynthesis or metabolism of indole-containing compounds, especially tryptophan-derived ones such as indole glucosinolate and auxin indole-3-acetic acid (IAA) (Supplemental Data Set 6). More detailed examination of the expression patterns for a comprehensive set of genes implicated in tryptophan metabolism revealed that some IAA biosynthesis genes, including *YUCCA1 (YUC1)* and *CYTOCHROME P450* genes (*CYP*s), were expressed at higher levels in the control condition compared to that in the MB-3 condition (Figure 3A; Supplemental Data Set 7A). Although not statistically significant, we also observed upregulation of a greater number of other *YUC*s in the control condition, suggesting that IAA biosynthesis is repressed by MB-3 treatment. Consistently, reverse transcription–quantitative polymerase chain reaction **(**RT–qPCR) analysis on cultured *hag3-1* protoplasts showed a similar reduction of *YUC1* transcripts (Supplemental Figure S4H) than that in WT protoplasts, supporting the notion that HATs regulate the transcriptional activation of auxin biosynthesis in protoplast reprogramming.

To test if IAA biosynthesis is required for cell cycle reinitiation, we examined whether blocking synthesis of this hormone impedes callus formation. As shown in Figure 3B and Supplemental Figure S5A, callus formation was strongly compromised in WT protoplasts treated with the IAA biosynthesis inhibitors yucasin and L-kynurenine (Kyn). Importantly, the inhibitory effect of yucasin was partially rescued by exogenous IAA application (Supplemental Figure S5B), suggesting that an optimum IAA level is required to induce reprogramming. We also examined if callus formation is affected in mutants compromised in IAA biosynthesis. As expected, multiple mutation of *YUC* genes using *yucQ* (*yuc3 yuc5 yuc7 yuc8 yuc9*) mutants (Chen et al., 2014) showed reduced callus formation efficiency (Figure 3C), although the single *yuc* mutants we tested did not show this phenotype (Supplemental Figure S5C). A similar reduction was also observed when using *CYP*s *family 79 subfamily B polypeptide 2* (*cyp79B2*) *cyp79B3* mutants (Figure 3D), which had been previously reported as showing reduced IAA biosynthesis activity (Zhao et al., 2002). Together, these data support the hypothesis that IAA biosynthesis promotes protoplast division.

Consistently, overexpression of *YUC1* in *LexA-VP16-estrogen receptor (XVE)-YUC1* protoplasts or application of low concentrations of IAA reproducibly increased callus formation efficiency (Figure 3E; Supplemental Figure S5, D and E), further substantiating the idea that the level of IAA is a key limiting factor for cell cycle reactivation. Interestingly, it seems that *YUC* expression within an appropriate dose range is needed to maximally promote cell cycle reinitiation. A low level of leaky *YUC1* expression under mock treatment or mild induction of the *YUC1* transgene by 0.1-μM β-estradiol (ED) in *XVE-YUC1* protoplasts was sufficient to increase callus formation efficiency compared to that in the WT, while strong overexpression of the *YUC1* transgene by 1- or 5-μM ED did not have this effect (Figure 3E; Supplemental Figure S5E). Furthermore, our data suggest that the timing of *YUC* expression is also important since induction of the *YUC1* transgene at Day 4 resulted in greater promotion of callus formation compared to induction at Day 0 (Figure 3E).

### Auxin biosynthesis promotes initial division of protoplasts by activating their auxin response

**Figure 4:**
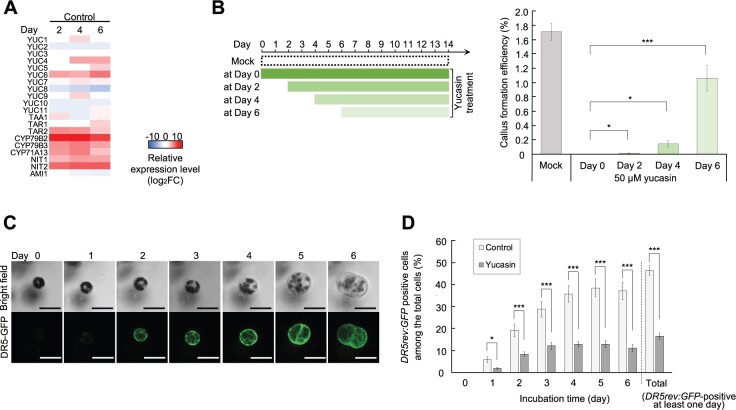
Early auxin biosynthesis is required to reinitiate cell division in leaf mesophyll protoplasts. A, Heat map of the transcriptional changes for representative IAA biosynthesis genes at Days 2, 4, and 6 in the control condition. Values (log_2_FC) are relative to Day 0. B, Callus formation efficiency of WT protoplasts treated with 50-μM yucasin at different time points. Left diagram shows the timing of yucasin treatment. Data are represented as mean ± sem. *n* = 15 from three biological replicates. ^*^P < 0.05, ^***^P < 0.001 (two-tailed Welch’s *t* test compared to yucasin treatment at Day 0). C, Time-lapse confocal microscopy images of a mesophyll protoplast that undergoes cell division at Day 5. Auxin response is visualized by *DR5rev:GFP* expression. D, Frequency of *DR5rev:GFP*-expressing (positive) protoplasts in the control or 50-μM yucasin condition among all tested *DR5rev:GFP* protoplasts. Error bars represent standard error. *n* = 9 region of interests (ROIs) containing 902 cells in total for control and 7 ROIs containing 730 cells in total for yucasin-treated protoplasts from 2 independent experiments. ^*^P < 0.05, ^***^P < 0.001 (two-tailed Welch’s *t* test). Scale bars are 30 μm (C).

Our results so far suggest that IAA biosynthesis prior to the initial cell division promotes callus formation from mesophyll protoplasts. Indeed, our RNA-seq data showed that IAA biosynthesis genes started to be upregulated before initial cell division, and the high expression levels lasted until Day 14 (Figures 3, A and 4, A; Supplemental Data Set 7). We, therefore, hypothesized that IAA biosynthesis promotes initial cell division. Notably, yucasin inhibited callus formation more strongly when it was added by Day 4, that is before most cells reinitiate the cell cycle, than when it was added later on (Figure 4B). This indicates that endogenous IAA production is essential for initial cell division, although it is also required for successive divisions.

To further investigate the role of IAA biosynthesis during early stages of protoplast reprogramming, we visualized the auxin response by time-lapse imaging of protoplasts carrying *DR5rev:GFP*, which expresses GFP via the auxin response element containing the *DR5* promoter (Ulmasov et al., 1997; Benková et al., 2003). In the control condition, we barely observed *DR5rev:GFP* expression in freshly isolated protoplasts, while many started to express detectable GFP signals from Days 2 to 4 (Figure 4, C and D), with nearly 50% of protoplasts showing *DR5rev:GFP* expression at least at one time point by Day 6. Importantly, we further found that IAA biosynthesis was required for activation of the auxin response, since yucasin treatment severely reduced the proportion of *DR5rev:GFP*-positive protoplasts (Figure 4D). We also note that *DR5rev:GFP*-positive cells in the control condition made up a large proportion of the protoplasts that divide, elongate or expand, whereas *DR5rev:GFP*-negative protoplasts in either the control or yucasin condition generally decreased in size (Supplemental Figure S6). These observations suggest that *DR5rev:GFP*-detectable auxin response promotes the early phases of developmental reprogramming, although that alone is not sufficient to complete the first cell division.

### Histone acetylation-mediated activation of PLTs is responsible for *YUC* upregulation

**Figure 5:**
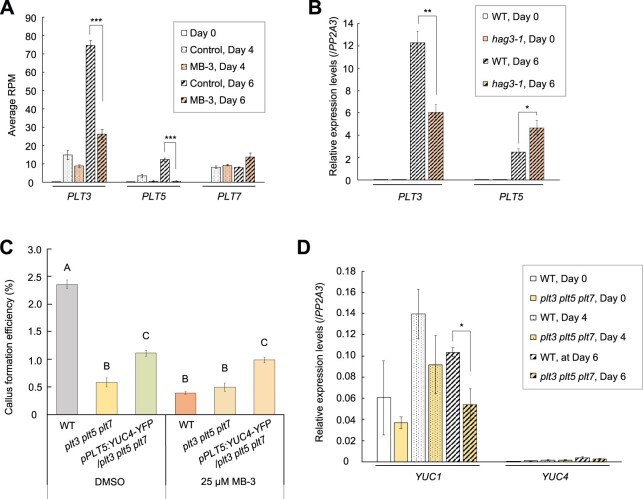
*PLT*s are upregulated by histone acetylation and transcriptionally activate auxin biosynthesis. A, Expression patterns of *PLT3*, *PLT5*, and *PLT7* at Days 0, 4, and 6 in the control and MB-3 conditions. RNA-seq data are represented as average reads per million (RPM) ± sem. *n* = 5 for Day 0 and *n* = 3 for Days 4 and 6. ^***^P < 0.001 (two-tailed Student’s *t* test). B, The expression levels of *PLT3* and *PLT5* in WT and *hag3-1* protoplasts cultured for 6 days. RT–qPCR data are represented as mean ± sem (*n* = 3). The expression levels are normalized to those of the internal control *PP2A3*. ^*^P < 0.05, ^**^P < 0.01 (two-tailed Student’s *t* test to WT). C, Callus formation efficiency of WT, *plt3 plt5 plt7*, and *pPLT5:YUC4-YFP/plt3 plt5 plt7* protoplasts. Half a microliter of DMSO with or without MB-3 was added to PCIM at the indicated final concentration at Day 0. Data are represented as mean ± sem. *n* = 10 from two biological replicates. Different letters indicate significant differences based on one-way ANOVA with post hoc Tukey’s HSD test (*P* < 0.001). D, Expression patterns of *YUC1* and *YUC4* at Days 0, 4, and 6 in WT and *plt3 plt5 plt7* protoplasts. RT–qPCR data are represented as mean ± sem (*n* = 3). The expression levels are normalized to those of the internal control *PP2A3*. ^*^P < 0.05 (two-tailed Student’s *t* test).

We next investigated how histone acetylation regulates YUC-mediated IAA biosynthesis. Given that *YUC* loci are not strongly acetylated in several regeneration contexts (Kim et al., 2018; Rymen et al., 2019), we searched for upstream transcriptional regulators that can be activated by histone acetylation. Among several transcriptional regulators that can induce *YUC* expression, we decided to focus on *PLETHORA*s (PLT3), PLT5, and PLT7, transcriptional activators of *YUC4* in wound-induced vascular regeneration (Radhakrishnan et al., 2020), since their loci are acetylated in the tissue culture condition (Kim et al., 2018) and the expression levels of their genes were sharply upregulated in cultured WT protoplasts compared to Day 0 (Figure 5A). We also found that the expression of *PLT3*, which was the most strongly upregulated *PLT*, was compromised by both MB-3 treatment and *hag3-1* mutation (Figure 5, A and B).

To explore the involvement of PLTs in protoplast reprogramming, we first examined whether PLTs are required for YUC-mediated callus formation. We observed that *plt3 plt5 plt7* mutants (Prasad et al., 2011) displayed reduced callus formation efficiency and, importantly, this impairment could be partially rescued by ectopic *YUC* expression driven by the *PLT5* promoter (Radhakrishnan et al., 2020) (Figure 5C). Consistently, RT–qPCR analysis showed that the upregulation of *YUC1* was less prominent in *plt3 plt5 plt7* protoplasts at Days 4 and 6 (Figure 5D), when *YUC1* expression was essential for the promotion of callus formation (Figure 3E). Unlike the case in vascular regeneration (Radhakrishnan et al., 2020), we did not detect significant alterations of *YUC4* expression by *plt* mutations (Figure 5D). We further observed that protoplasts isolated from *plt3 plt5 plt7* and *pPLT5:YUC4-YFP/plt3 plt5 plt7* plants were less sensitive to MB-3 treatment compared to WT protoplasts (Figure 5C). Notably, *pPLT5:YUC4-YFP/plt3 plt5 plt7* protoplasts showed better callus formation efficiency than *plt3 plt5 plt7* protoplasts even in the presence of MB-3 (Figure 5C), supporting the notion that PLT-YUC activation is the core downstream pathway regulated by histone acetylation. Collectively, these results suggest that PLTs are transcriptionally upregulated through histone acetylation and promote protoplast division by increasing *YUC* expression.

### Auxin biosynthesis is required to transcriptionally activate G2/M phase genes

**Figure 6:**
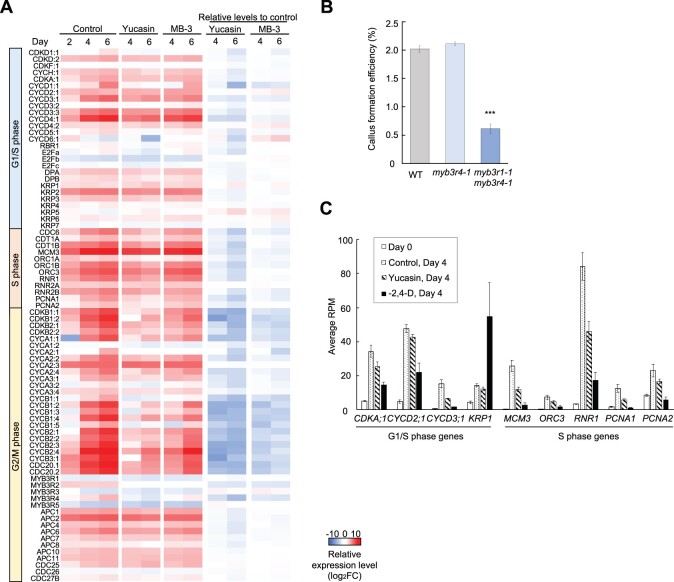
Auxin biosynthesis is required to transcriptionally activate the G2/M phase genes through MYB3R1 and MYB3R4. A, Heat map representing the transcriptional changes for genes encoding core cell cycle regulators at G1/S, S, and G2/M phases at Days 2, 4, and 6. The left three columns show expression levels in the control, 50-μM yucasin and 50-μM MB-3 conditions as values normalized (log_2_FC) to Day 0. The “Relative levels to control” columns show the normalized expression levels in the yucasin or MB-3 condition compared to the control condition for respective time points as relative values (log_2_FC). Gene sets were selected based on Kalve et al. (2014). B, Callus formation efficiency of WT, *myb3r4-1*, and *myb3r1-1 myb3r4-1* protoplasts. Data are represented as mean ± sem. *n* = 20 from four biological replicates for the WT and *myb3r1-1 myb3r4-1* and *n* = 15 from three biological replicates for *myb3r4-1*. ^***^P < 0.001 (two-tailed Student’s *t* test compared to WT). C, Examples of the G1/S phase-related genes that were differently expressed in the –2,4-D condition compared to that in the control and yucasin conditions at Day 4. RNA-seq data are represented as average RPM ± sem. *n* = 6 for Day 0 and the control condition at Day 4 and *n* = 3 for the yucasin and –2,4-D conditions at Day 4.

To further reveal how auxin promotes resumption of the mitotic cell cycle, we compared expression patterns of core cell cycle regulators under control and yucasin conditions by RNA-seq (Figure 6A; Supplemental Data Set 8A). Previous studies have shown that freshly isolated leaf mesophyll protoplasts reside at the G1 phase and enter the S phase only upon phytohormone application (Zhao et al., 2001). Consistently, genes functioning during the G1 to S phase, including *D-type CYCLIN*s (*CYCD*s) and *MINICHROMOSOME MAINTENANCE 3* (*MCM3*), showed upregulation from Day 2 in the control condition. In agreement with our observation that the timing of initial cell division peaks around Day 6, genes functioning during the G2 to M phase, such as *B-type CYCLIN-DEPENDENT KINASE*s, *B-type CYCLIN*s, *CELL DIVISION CYCLE 20.1* (*CDC20.1*), and *CDC20.2*, were upregulated particularly at Days 4 and 6. Strikingly, in the 50-μM yucasin condition, the expression of G1/S genes was comparable to that in the control condition, while many *G2/M* genes were clearly suppressed (Figure 6A). This notion was further confirmed when a larger set of genes that show transcriptional activation in S phase or G2/M phase were examined (Kobayashi et al., 2015) (Supplemental Figure S7A; Supplemental Data Set 8, B and C), suggesting that auxin biosynthesis is required to transcriptionally activate the G2/M phase genes. We also observed similar transcriptional trends in the 50-μM MB-3 condition (Figure 6A; Supplemental Figure S7A), supporting our hypothesis that one of the key downstream pathways regulated by histone acetylation is the IAA biosynthesis-dependent G2/M progression. Intriguingly, yucasin or MB-3 treatment had little impact on the transcription of photosynthetic genes (Supplemental Data Set 1), suggesting that this aspect of cellular dedifferentiation is regulated by mechanisms independent from histone acetylation and IAA biosynthesis.

The identification of a number of G2/M genes transcriptionally targeted by auxin biosynthesis and histone acetylation suggests that some master regulators of these genes are involved in this regulation. Previous studies have shown that MYB3R4, together with MYB3R1, functions as a transcriptional activator of G2/M genes and their peak in activity at the G2/M phase is regulated at transcriptional and/or posttranslational levels (Haga et al., 2007; Haga et al., 2011; Kobayashi et al., 2015; Yang et al., 2021). Indeed, our RNA-seq data showed that the expression of *MYB3R4* was sharply upregulated from Days 2 to 6 in the control but not in either the yucasin or MB-3 condition (Figure 6A), implying that auxin biosynthesis and histone acetylation regulate cell cycle progression by transcriptionally upregulating *MYB3R4*. To test whether these activator MYB3Rs regulate cell cycle reentry in protoplasts, we isolated protoplasts from *myb3r4-1* or *myb3r1-1 myb3r4-1* mutants and tested their callus formation efficiencies. As shown in Figure 6B, *myb3r1-1 myb3r4-1* mutants, in which overall expression of *G2/M* genes are strongly repressed (Haga et al., 2007; Haga et al., 2011), had severely impaired callus formation efficiencies, indicating that these activator MYB3Rs are required for cell cycle reinitiation in protoplasts.

The discovery that auxin biosynthesis is critical for protoplast cell cycle reinitiation is surprising considering that PCIM contains exogenous auxin, 2,4-D, which is indispensable for initial cell division (Carle et al., 1998). Previous studies have indicated that exogenous auxin regulates the G1/S transition at both transcriptional and posttranslational levels in protoplasts (Carle et al., 1998; Pasternak et al., 2000), raising the possibility that exogenously supplied 2,4-D and endogenously produced IAA regulate different phases of the cell cycle to reinitiate cell division in protoplasts. To test this hypothesis, we compared the transcriptional activation of S phase and G2/M phase genes in control, yucasin and 2,4-D-omitted (–2,4-D) culture conditions. Our data show that many of the genes essential for S phase progression, such as *CYCD2;1*, *CYCD3;1*, and *MCM3*, were clearly suppressed in the –2,4-D condition compared to that in the control and yucasin conditions, whereas G2/M phase genes showed similar levels of hypo-activation in both the yucasin and –2,4-D conditions (Figure 6C, Supplemental Figure S7B and Supplemental Data Set 8, D and E). We also found that *KIP-RELATED PROTEIN 1*, which encodes an inhibitor for CDKA/CYCD complexes, was transcriptionally activated in the –2,4-D condition (Figure 6C), further suggesting repression of the G1/S transition. These results, therefore, suggest that exogenous 2,4-D and endogenous IAA may play distinct roles during the initial division of protoplasts, where they transcriptionally activate the S phase and G2/M phase progression, respectively.

### An ARF7/ARF19 and IAA3/IAA18-mediated auxin signaling pathway regulates cell cycle reinitiation in protoplasts

**Figure 7:**
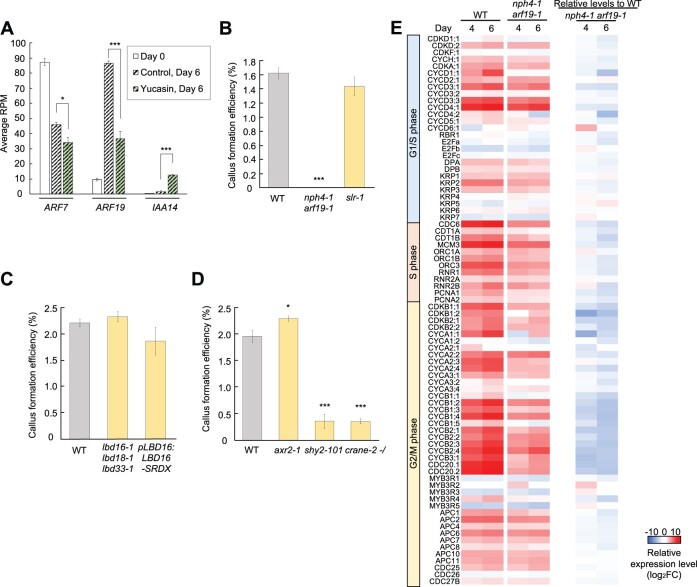
ARF7/ARF19 and IAA3/IAA18-mediated auxin signaling pathway drives cell cycle reinitiation in leaf mesophyll protoplasts. A, Expression patterns of *ARF7*, *ARF19*, and *IAA14* at Days 0 and 6 in the control and yucasin conditions. RNA-seq data are represented as average RPM ± sem. *n* = 5 for Day 0 and *n* = 3 for Days 4 and 6. ^*^P < 0.05, ^***^P < 0.001 (two-tailed Student’s *t* test). B, Callus formation efficiency of WT, *nph4-1 arf19-1*, and *slr-1* protoplasts. Data are represented as mean ± sem. *n* = 25 from five biological replicates for the WT and 20 from four biological replicates for the others. ^***^P < 0.001 (two-tailed Welch’s *t* test compared to WT). C, Callus formation efficiency of WT, *lbd16-1 lbd18-1 lbd33-1*, and *pLBD16:LBD16-SRDX* protoplasts. Data are represented as mean ± sem. *n* = 20 from four biological replicates for the WT and 15 from three biological replicates for the others. No statistical difference was detected (two-tailed Welch’s *t* test compared to WT). D, Callus formation efficiency of WT, *shy2-101*, *axr2-1*, and *crane-2* protoplasts. Since heterozygous and homozygous *crane-2* are morphologically indistinguishable, both genotypes were mixed for protoplast preparation. Data are represented as mean ± sem. *n* = 25 from five biological replicates for the WT and 15 from three biological replicates for the others. ^*^P < 0.05, ^***^P < 0.001 (two-tailed Welch’s *t* test compared to WT). E, Heat map representing the transcriptional changes for genes encoding core cell cycle regulators at G1/S, S, and G2/M phases at Days 4 and 6. The left two columns show expression levels in the WT and *nph4-1 arf19-1* protoplasts as values normalized (log_2_FC) to Day 0 of respective genotype. The “Relative levels to WT” columns show the normalized expression levels in *nph4-1 arf19-1* compared to that in the WT for respective time points as relative values (log_2_FC). Gene sets were selected based on Kalve et al. (2014).

Having established the central roles of auxin in protoplast reprogramming, we next sought to investigate how auxin-derived information evokes cell division. One possibility is that auxin activates the ARF-mediated signaling pathways to induce downstream gene expression. As reported previously, auxin is also critical for other forms of cellular reprogramming in plants, including pluripotent callus formation from explants in tissue culture (Christianson and Warnick, 1983). In Arabidopsis, auxin promotes cell cycle reactivation in pericycle cells on callus induction medium (CIM), and this is mediated by ARF7, ARF19, IAA14, and LATERAL ORGAN BOUNDARIES DOMAINs (LBDs) (Iwase et al., 2011; Fan et al., 2012; Lee et al., 2017). Our RNA-seq data showed that many of these auxin signaling regulators were sharply upregulated before initial cell division of protoplasts (Supplemental Figure S8A and Supplemental Data Set 9A), implying that they also participate in cell cycle reinitiation from differentiated leaf cells. Additionally, we found that the expression of *ARF7* and *ARF19*, which encode activators of auxin signaling, was reduced by yucasin treatment and, in contrast, the expression of *IAA14*, which encodes a repressor, was increased (Figure 7A; Supplemental Data Set 9A), suggesting that these ARFs, IAA14, and LBDs are involved in the downstream signaling pathways of IAA biosynthesis-mediated protoplast division. A loss-of-function mutant for ARF7, *non-phototrophic hypocotyl 4-1* (*nph4-1*) (Okushima et al., 2005), indeed displays defects in the auxin response in mesophyll protoplasts (Wang et al., 2005) and, consistently, single or double mutants for *ARF7* and *ARF19* were impaired in callus formation from protoplasts (Figure 7B; Supplemental Figure S8B). Intriguingly, however, a gain-of-function mutant for *IAA14*, *solitary root-1* (*slr-1*) (Fukaki et al., 2002), and two loss-of-function mutants for *LBD*s, *pLBD16:LBD16-SRDX*, and *lbd16-1 lbd18-1 lbd33-1* (Goh et al., 2012b), made callus from protoplasts with a similar efficiency as the WT, despite displaying severe defects in callus formation from hypocotyl explants in tissue culture (Iwase et al., 2011) (Figure 7, B and C; Supplemental Figure S8C). These results indicate that reprogramming from differentiated leaf cells is regulated by distinct auxin signaling pathways.

To further identify the auxin signaling components responsible for protoplast reprogramming, we selected other Aux/IAA candidates that can act by interacting with ARF7 and ARF19. Presumably, these Aux/IAAs repress ARF activities in intact leaves, but they might be downregulated in protoplasts to activate ARF-mediated auxin signaling. We thus decided to focus on those that have expression magnitudes comparable with those of *ARF7* and/or *ARF19* in leaves, show downregulation during protoplasting and/or subsequent culturing, and show binding to these ARFs (Piya et al., 2014) (Supplemental Figure S8, D and E and Supplemental Data Set 9B). Among them, we found that gain-of-function mutants for *IAA3* and *IAA18*, *suppressor of hy2-101* (*shy2-101*) (Goh et al., 2012a) and *crane-2* (Uehara et al., 2008), respectively, displayed strongly reduced callus formation from protoplasts (Figure 7D). The expression of *IAA7* was also strongly downregulated during protoplasting but its interaction was detected only with ARF19 and not with ARF7 (Piya et al., 2014) (Supplemental Figure S8E). The gain-of-function mutant for *IAA7*, *auxin resistant 2-1* (*axr2-1*) (Nagpal et al., 2000), did not cause obvious defects in callus formation (Figure 7D), suggesting that IAA7 does not play major roles in protoplast reprograming. Collectively, our results suggest that IAA3 and IAA18, together with ARF7 and ARF19, mediate the auxin response to drive cell cycle reinitiation in protoplasts.

Having identified the key auxin signaling components, we next sought to investigate how the ARF7/ARF19-dependent pathway regulates cell cycle reinitiation; therefore, we compared the expression of cell cycle genes between WT and *nph4-1 arf19-1* protoplasts (Figure 7E). Interestingly, we found that the expression patterns of cell cycle genes in *nph4-1 arf19-1* protoplasts resembled those in yucasin-treated WT protoplasts since many *G2/M* genes were clearly downregulated in *nph4-1 arf19-1* protoplasts (Figures 6, A and 7, E; Supplemental Figures S7, A and S9; Supplemental Data Sets 8 and 10). Notably, the transcriptional activation of *MYB3R4* was suppressed in *nph4-1 arf19-1* protoplasts, likely accounting for the global downregulation of *G2/M* genes (Figure 7E). As we observed for yucasin-treated protoplasts, the expression of *G1/S* genes was generally less affected in *nph4-1 arf19-1* protoplasts (Figures 6, A and 7, E; Supplemental Figures 7, A and S9; Supplemental Data Sets 8 and 10). These data, therefore, support that ARF7 and ARF19 play profound roles in transcriptionally activating G2/M phase genes through the upregulation of *MYB3R4* downstream of IAA biosynthesis.

## Discussion

**Figure 8:**
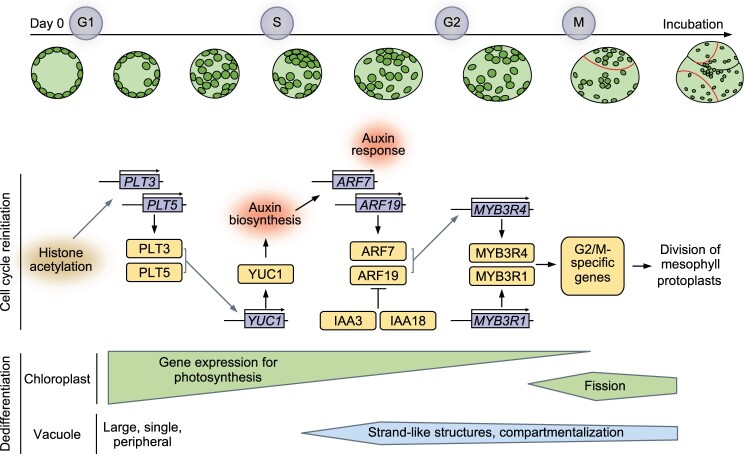
Hypothetical model describing the molecular mechanism of developmental reprograming in leaf mesophyll protoplasts. Freshly isolated Arabidopsis leaf mesophyll protoplasts undergo dramatic developmental reprograming and produce pluripotent callus when cultured in the presence of auxin and cytokinin. Histone acetylation is required to reinitiate the mitotic cell cycle and one of the key downstream pathways regulated by histone acetylation is transcriptional activation of PLTs. PLTs are required for the induction of *YUC1* encoding an auxin biosynthesis gene. Endogenously produced auxin, IAA, in turn increases auxin response in protoplasts, leading to the activation of ARF7/ARF19 and IAA3/IAA18-mediated auxin signaling. ARF7 and ARF19 are responsible for the transcriptional upregulation of MYB3R4, which then activates the transcription of G2/M phase genes together with MYB3R1 to complete cell division. Another key feature of developmental reprograming that protoplasts undergo at an early stage of culture is cellular dedifferentiation, which is marked, for instance, by drastic downregulation of photosynthetic genes and upregulation of chloroplast fission genes. These transcriptional changes are independent of histone acetylation and auxin biosynthesis. In addition, a large, single vacuole, typical of differentiated cells, becomes compartmentalized and starts to resemble what is found in proliferating or elongating cells.

In this study, we demonstrate that one of the key factors that permit reprograming of differentiated plant cells is the activation of auxin biosynthesis. Although cellular dedifferentiation seems to already start during the process of protoplast preparation to some degree (Supplemental Figure S2; Supplemental Data Set 1), our data show that endogenously produced IAA is indispensable for increasing the auxin response in isolated protoplasts, thereby inducing the expression of *G2/M* genes to reinitiate the cell cycle (Figure 8). Notably, auxin biosynthesis is essential for the first cell division while it is less critical for successive divisions (Figure 4B). Since differentiated cells should be equipped with mechanisms to prevent ectopic cell proliferation (Ikeuchi et al., 2015), it is plausible that cell cycle reinitiation in protoplasts requires some unique mechanisms in addition to those functioning during subsequent cell proliferation, with the latter being more similar to those at play during normal development. Previous studies, for instance, have shown that differentiating leaf cells accumulate regulators, such as DA1 and MEDIATOR 25, important for repressing proliferation and promoting cellular growth (Kalve et al., 2014). In parallel, they shut down the expression of many cell cycle activators such as *CYCD4*s and *ANAPHASE COMPLEX 10* through epigenetic mechanisms (Ikeuchi et al., 2015; Candeale et al., 2014). Differentiating cells, additionally, develop physical properties such as thickened cell walls and enlarged vacuoles that may inhibit cell division. Restarting the cell cycle should therefore require the removal of these negative factors and/or induction of some potent activators that can drive cell cycle reentry. In the case of mesophyll protoplasts, auxin biosynthesis induces the transcription of G2/M phase genes likely through MYB3R4 and MYB3R1 (Figure 6), suggesting that these MYB3Rs serve as key re-activators of cell division. These MYB3Rs appear to be dispensable for mesophyll cell proliferation in intact leaves (Haga et al., 2011), so they might be required to induce cell division specifically in differentiated cells where other factors promoting cell cycle progression are possibly inactivated. How auxin biosynthesis regulates MYB3R4/1-mediated pathways is an important question. Our data suggest that auxin biosynthesis transcriptionally activates *MYB3R4* through the ARF7 and ARF19-mediated pathway (Figures 6, A and 7, E). We speculate that MYB3R4, as well as MYB3R1, might also be subject to posttranslational regulation since phosphorylation and temporal nuclear shuttling of MYB3Rs are central for their functions during mitosis (Chen et al., 2017; Yang et al., 2021).

Accumulating evidence suggests that IAA biosynthesis is a prerequisite for several modes of plant regeneration. Local production of auxin, for instance, is required for de novo adventitious root formation from vascular cells in leaf explants (Chen et al., 2016), reconstruction of the root meristem following its excision (Matosevich et al., 2020) and vascular reconnection (Radhakrishnan et al., 2020). Our genetic and pharmacological data suggest that the same requirement holds true for the reprograming of differentiated leaf mesophyll cells (Figure 3; Supplemental Figure S5), further highlighting the critical role of IAA biosynthesis as a core regulatory module for cellular reprogramming in plants. On the other hand, a recent study demonstrated that IAA biosynthesis has a negative effect on the acquisition of shoot regenerative competence in tissue culture (Ohbayashi et al., 2022), suggesting the diverse roles of IAA biosynthesis in plant cell reprogramming. We also demonstrate that there is a key difference in how IAA biosynthesis is activated since our data suggest that PLTs increase IAA biosynthesis through transcriptional activation of *YUC1* (Figure 5), not *YUC4* as in vascular regeneration (Radhakrishnan et al., 2020). What differences, for instance in cell types being reprogrammed and/or physiological environments surrounding those cells, are reflected in such diverse pathways is an important question to be addressed in future studies. Given that only a limited proportion of mesophyll protoplasts reinitiate the cell cycle, another critical question is when and how cells are directed to undergo reprogramming and, importantly, how strictly this decision is linked with cell-autonomous auxin production. Microscopic observation of protoplasts expressing fluorescently labeled IAA biosynthetic enzymes should provide the spatiotemporal information necessary to distinguish the events happening in dividing cells from those occurring in nondividing cells. Single-cell RNA-seq analysis will also be a great tool to investigate transcriptional differences between dividing and nondividing cells.

Given that exogenously supplied 2,4-D is essential to form callus from protoplasts (Supplemental Table S1), it clearly serves as another strong driver of cell cycle reinitiation in protoplasts. Interestingly, our data suggest that 2,4-D and endogenously produced IAA may activate distinct phases of the cell cycle to reinitiate protoplast division (Figure 6C; Supplemental Figure 8B; Supplemental Data Set 8, D and E). This is consistent with earlier reports that exogenous 2,4-D is required during the initial steps of protoplast culture (Carle et al., 1998), while IAA biosynthesis seems to become important later (Pasternak et al., 2002) (Figure 3E). How these two types of auxin may activate different cell cycle phases is not clear at this point. Our data suggest that the ARF7- and ARF19-mediated auxin signaling pathway can confer IAA signaling to activate G2/M phase genes during protoplast reprogramming (Figure 7E; Supplemental Figure S9), although our data do not exclude the possibility that ARF7 and ARF19 also mediate 2,4-D signaling. An interesting question that should be addressed in future work is how these (and potentially other) auxin response factors (ARFs) might be activated by specific auxin species. Previous studies suggest that 2,4-D and IAA are transported differently through plasma membranes and tonoplasts (Delbarre et al., 1996; Ranocha et al., 2013) and are metabolized differently (Eyer et al., 2016), implying that some of these biochemical properties have previously uncharacterized physiological implications that can activate a specific auxin response pathway. Our data are also consistent with the idea that transcriptional activation of *G2/M* genes requires higher levels of auxin than those required for G1/S gene activation. In this scenario, exogenously supplied 2,4-D might be sufficient to drive the G1/S transition but both 2,4-D and endogenously produced IAA are required for the G2/M transition.

Alternatively, stimulation of the IAA biosynthetic pathway itself might play a crucial role in protoplast division. For instance, the YUC cofactor flavin adenine dinucleotide is oxidized and produces hydrogen peroxide (H_2_O_2_) when the IAA precursor indole 3-pyruvate is deficient (Dai et al., 2013). H_2_O_2_ has been demonstrated to either promote or inhibit cell cycle reinitiation in protoplasts depending on the timing of its production and subcellular localization (De Marco and Roubelakis-Angelakis, 1996; Tiew et al., 2015). Since *YUC* genes need to be upregulated at an appropriate dose and time during protoplast culture (Figure 3E), activation of the IAA biosynthetic pathway may contribute to protoplast reprogramming through the production of its byproducts such as H_2_O_2_. It is also worth considering the possibility that overall regulation of indole-containing compound metabolism affects protoplast reprogramming, since our RNA-seq data show the striking upregulation of the indole metabolism-related genes that may not act in the IAA biosynthetic pathway (Supplemental Data Set 6). Investigating the potential contribution of these nonhormonal metabolites may help uncover a new regulatory mechanism underlying plant cellular reprogramming.

While this study provides an important molecular insight into the developmental reprogramming of mesophyll protoplasts, we should note that other signaling pathways also likely play roles in triggering this process. Our transcriptomic data suggest that dedifferentiation starts during protoplast isolation (Supplemental Figure S2; Supplemental Data Set 1) and this process appears to proceed independently of histone acetylation and IAA biosynthesis (Supplemental Data Set 1). What drives these early dedifferentiation steps is an open question to be investigated in future studies. In addition, we found that genes induced in a histone acetylation-dependent manner are enriched for those involved in the defense responses such as glucosinolate biogenesis and the jasmonic acid (JA) response (Figure 2C; Supplemental Data Set 6A). It is well known that cell wall perturbation by biotic and abiotic stress, caused for example by pathogen attack or wounding, activates the expression of defense response genes (Bacete et al., 2018) and JA production (Mielke and Gasperini, 2019). It is thus possible that similar stress-sensing mechanisms are activated by the removal of cell walls during protoplast isolation, and that these also contribute to protoplast reprogramming. It will be interesting to investigate how cell wall-derived signals are transduced in mechanically injured cells and affect developmental programs during both protoplast reprogramming and other forms of regeneration that should also be characterized by similar but more local cell wall perturbations.

## Materials and methods

### Plant materials and growth conditions

*Arabidopsis thaliana* (L) Heynh. ecotype Columbia-0 (Col-0) was used as the WT. For T-DNA-inserted lines, see the “Accession numbers” described below. *plt3 plt5 plt7* (Prasad et al., 2011), *nph4-1 arf19-1* (Okushima et al., 2005), *arf7-2 arf19-5* (Goh et al., 2012a), *slr-1* (Fukaki et al., 2002), *axr2-1* (Nagpal et al., 2000), *shy2-101* (Goh et al., 2012b), *crane-2* (Uehara et al., 2008), *pPLT5:YUC4-YFP*/*plt3 plt5 plt7* (Radhakrishnan et al., 2020), *pVHP1:VHP1-mGFP* (Segami et al., 2014), and *DR5rev:GFP* lines (Benková et al., 2003) were previously described. All these mutants and transgenic plants were in the Col-0 background. Seeds were sterilized with 70% ethanol for 1 min and 20% chlorine bleach (Kao) for 10 min, and then rinsed with autoclaved water 3 times. After being soaked in water at 4°C for 2–4 days, seeds were sown on germination medium (GM) (Supplemental Table S1) at a density of 33 seeds per 90-mm diameter and 25-mm thick polystyrene dish (Kord-Valmark). Plants were grown at 22°C under continuous light (30–40 μmol m^−2^ s^−1^) in a growth chamber (Sanyo).

### Transgenic plants

To construct the *pCAB3:H2A-eGFP* vector, the *CAB3* promoter 1,537-bp upstream of the ATG start codon was amplified from genomic DNA by polymerase chain reaction (PCR). The PCR products were purified and cloned into the *pDONRP4P1r* vector (Thermo Fisher Scientific, Waltham, MA, USA). The promoter fragment was then assembled with GAL4 into the *pB-9FH2A-UAS-7m24GW* destination vector in a multi-site gateway reaction to create an activator line construct using the LR Clonase II+ (Thermo Fisher Scientific). This destination vector contains a H2A-encoding sequence fused to eGFP and driven by the repetitive UAS promoter, as described by Fendrych et al. (2014). To construct the *pHAG1:HAG1-GFP* vector, the *HAG1* promoter 1,987-bp upstream of the ATG start codon was amplified from genomic DNA, purified and cloned into the *pDONRP4P1r* vector (Thermo Fisher Scientific). The *HAG1* coding sequence was amplified from complementary DNA (cDNA) by PCR, purified and cloned into the *pENTR/D-TOPO* vector (Thermo Fisher Scientific). The promoter and coding sequence fragments were then assembled with GFP into the *R4pGWB504* destination vector in a multi-site gateway reaction using Gateway LR Clonase II (Thermo Fisher Scientific). To construct the *XVE-YUC1* vector, the *YUC1* coding sequence was amplified from cDNA by PCR. The PCR products were purified and cloned into the *pENTR/D-TOPO* vector (Thermo Fisher Scientific). The cDNA fragments were then cloned into the modified *pER8* plasmid containing a Gateway cassette (*pER8-GW*) (Favero et al., 2020) using Gateway LR Clonase II (Thermo Fisher Scientific). For plant transformation, the plasmids were introduced into *Agrobacterium tumefaciens* (strain GV3101) by electroporation and transformed into Arabidopsis Col-0 WT plants (for *pCAB3:H2A-eGFP* and *XVE-YUC1*) or *hag1-1* heterozygous plants (for *pHAG1:HAG1-GFP*) using the floral dip method (Clough and Bent, 1998). As for *pHAG1:HAG1-GFP/hag1-1* plants, after the selection of single insertion lines of *pHAG1:HAG1-GFP* at T2, the lines homozygous for the *hag1-1* mutation were further selected by PCR genotyping. Lines homozygous both for *pHAG1:HAG1-GFP* and *hag1-1* were then selected at T3. The primer sequences used for the constructs described above are listed in Supplemental Table S2.

### Chemical compounds

2,4-D (Cas 94-75-7, Sigma, St. Louis, MO, USA), C646 (Cas 328968-36-1, Sigma) (Bowers et al., 2010), ED (Cas 50-28-2, Wako), garcinol (Cas 78824-30-3, Focus biomolecules) (Balasubramanyam et al., 2004), kinetin (Kin) (Cas 525-79-1, SIGMA), Kyn (Cas 2922-83-0, TCI) (He et al., 2011), γ-butyrolactone (MB-3) (CAS 778649-18-6, Abcam, Cambridge, UK) (Biel et al., 2004), TDZ (Cas 51707-55-2, Wako), 5-(4-chlorophenyl)-4H-1,2,4-triazole-3-thiol (yucasin) (CAS 26028-65-9, Wako) (Nishimura et al., 2014), and 6-(γ,γ-dimethylallylamino)purine (2-iP) (CAS 2365-40-4, Sigma) were dissolved in dimethyl sulfoxide (DMSO) and sterilized by filtration. IAA (Cas 6505-45-9, Wako) was dissolved in ethanol and sterilized by filtration. FDA (Cas 596-09-8, DOJINDO) was dissolved in DMSO and used without sterilization. All chemicals were stored at −20°C.

### Protoplast isolation and callus induction

Protoplasts were isolated following Damm and Willmitzer (1988) with modifications. All processes were performed at room temperature (22°C –25°C) unless otherwise specified. The first to fifth rosette leaves of plants at 23 or 24 days after sowing (DAS) were aseptically harvested. After carefully removing petioles, the leaves were chopped into strips with a scalpel (Akiyama) in 0.5-M mannitol. After 1-h maceration in 0.5-M mannitol under dim light, leaf strips from 100 to 150 leaves were transferred into a 35-mm polystyrene petri dish (FALCON) and soaked in 3 mL of Digestion Cocktail (Supplemental Table S1). For cell wall digestion, leaf strips were gently shaken horizontally on a Shake-LR (TAITEC) for 3 h in the dark. Protoplasts were then separated from leaf tissues by filtration through a 40-μm nylon Cell Strainer (FALCON) with gentle pipetting using a Komagome type pipette (IWAKI). The filtrate was collected into a 12-mL culture tube with a conical bottom (Simport), diluted with 1/2 volume of 0.2-M CaCl_2_ and centrifuged for 5 min at 60*g* in a swinging bucket rotor (CF16RN, HITACHI) with the slowest acceleration and no brake. The pellet was resuspended in 5 mL of Wash Medium 1 (Supplemental Table S1) and centrifuged for 3 min at 40*g*. The resulting pellet was resuspended in 5 mL of Wash Medium 2 (Supplemental Table S1) and centrifuged again. The pellet was subsequently resuspended in 5 mL of 0.5-M mannitol and centrifuged again. The protoplasts were finally resuspended in fresh 0.5-M mannitol and placed on ice for 40 min–1 h under dim light. After calculating cell density with a hemocytometer (Sunlead Glass Corp.), the protoplasts were warmed to room temperature and their density was adjusted to 4.8–5.0 × 10^5^ cells mL^−1^ with 0.5-M mannitol.

Isolated protoplasts were cultured using the protocol modified from Damm and Willmitzer (1988), Masson and Paszkowski (1992), and Chupeau et al. (1993). For embedding protoplasts in the sodium alginate gel, the protoplast solution was gently mixed with an equal volume of Sodium Alginate Solution (Supplemental Table S1), and 200 μL of this solution was then dropped onto a CaCl_2_ Plate (Supplemental Table S1) using a truncated tip. Sodium alginate gels, each containing ∼4.8–5.0 × 10^4^ cells, were solidified into a piece of gel for 1 h at room temperature under dim light. Five pieces of gels were subsequently transferred into a well of a 6-well microplate (IWAKI) or a 35-mm petri dish (FALCON) filled with 4 mL of PCIM (Supplemental Table S1). The plates and dishes were then sealed with surgical tape and parafilm, respectively, and embedded protoplasts were aseptically cultured at 22°C in the dark.

Callus formation efficiency was evaluated at Day 14 unless otherwise specified. Calli embedded in each sodium alginate gel were counted under a dissection microscope (M165 FC, Leica) at 2.5 × magnification. Callus formation efficiency was calculated as a percentage of the number of calli in the number of protoplasts initially contained in one sodium alginate gel. The reproducibility was confirmed in at least three biological replicates, using protoplasts isolated from different sets of plants in three independent experiments. As the control experiments in chemical treatment assays, protoplasts of corresponding genotypes were treated with the equivalent volume of solvent (DMSO or ethanol) to confirm that their callus formation efficiencies are not affected by these treatments.

### Induction of shoot formation from protoplast-derived callus

After incubating in PCIM for 14 days, calli-containing gels were transferred to 4 mL of CGM (Supplemental Table S1) and cultured at 22°C under continuous dim light (10–12 μmol m^−2^ s^−1^). CGM was refreshed every 2 weeks. For induction of de novo shoot formation, calli were isolated from the gels by shaking them in Citrate Solution (Supplemental Table S1) for 1–2 h, which chelates calcium ions and dissolves the gel. Isolated calli were washed twice with liquid Gamborg B5 medium and cultured on SIM (Supplemental Table S1) at 22°C under continuous light (22–28 μmol m^−2^ s^−1^).

### Callus induction from etiolated hypocotyls

To induce callus from tissues, seeds were sown on GM for Tissue Culture (Supplemental Table S1) and grown at 22°C in the dark for 7 days. Hypocotyls of etiolated seedlings were excised into around 5-mm-long explants using a scalpel and incubated on CIM (Supplemental Table S1) for 21 days at 22°C under continuous light (22–28 μmol m^−2^ s^−1^). The reproducibility was confirmed in two biological replicates.

### Viability test

To test the viability of protoplasts, FDA was added to an aliquot of protoplast solution at the final concentration of 1 mg L^−1^ and incubated for 10 min. The stained cells were mounted onto a microscope slide and observed with a fluorescence microscope (BX51, OLYMPUS).

### Live imaging of protoplast reprograming

The microscopic observation method for tracking identical protoplasts was developed based on Hall et al. (1995) and Dovzhenko et al. (1998). To immobilize the protoplast-containing gels on the bottom of a 35-mm petri dish, a polypropylene grid (1.7 × 1.7-mm mesh size, NIP), cut into 25 × 25-mm sections, was placed in a CaCl_2_ plate and the protoplast solution was solidified on the mesh. The gel was subsequently fixed to the bottom of a petri dish using the attached grid and an additional four gels were placed in the same dish to keep the number of protoplasts within a well constant as in our standard culture condition. These gels were cultured with 4 mL of PCIM at 22°C in the dark.

Time-lapse imaging was performed using a confocal microscope (TCS-SP5, Leica) with a water immersion lens (HC FLUOTAR L 25×/0.95 W VISIR, Leica). The protoplast-containing petri dishes were placed on the microscopic stage at each time of observation. Region of interests was manually tracked based on the coordinates (*x, y, z*) recorded on the first day. Z-stacked images were taken at 6-μm intervals to maximize the number of protoplasts that can be tracked. Each observation was performed within 1-h per dish to avoid excessive light exposure of protoplasts. Images were processed and analyzed with Fiji version 2.0.0 (https://imagej.net/Fiji) and Microsoft Excel. The reproducibility was confirmed in at least two biological replicates.

### Transcriptome analysis

RNA was extracted from ∼1 g (in fresh weight) of rosette leaves or 1.0–1.5 × 10^6^ protoplasts that were prepared from 23-DAS plants. Freshly isolated protoplasts were collected as a pellet in a 2-mL tube by centrifugation for 1 min at 3,200*g* (MX-305, TOMY) and stored at −80°C. To collect cultured protoplasts embedded in the sodium alginate gel, 25 gels per sample were soaked in 25 mL of citrate solution and protoplasts were released by gentle shaking for 1 h and then collected by centrifugation. Three biological replicates were prepared for each time point. Total RNA was isolated using an RNeasy plant mini kit (Qiagen, Hilden, Germany). Isolated RNA was then subjected to library preparation using a Kapa stranded mRNA sequencing kit (Kapa Biosystems, London, UK) with NEBNext Multiplex Oligos for Illumina (New England Biolabs, Ipswich, MA, USA) as adapters and Agencourt AMPure XP (Beckman Coulter, Brea, CA, USA) beads instead of KAPA Pure Beads. Single-end sequencing was performed on an Illumina NextSeq500 platform. Mapping was carried out using Bowtie version 0.12.9 (Langmead and Salzberg, 2012). Over 50% of the reads were uniquely mapped to the TAIR10 Arabidopsis genome, resulting in 5–17 million mapped reads per sample. DEGs were identified using the edgeR package (Robinson et al., 2010) in R/Bioconductor (https://www.r-project.org/) after normalization of total read counts with the trimmed mean of M-values method. DEGs were defined as those that showed |log_2_FC| > 2 in transcript levels (*P* < 0.01 and FDR < 0.01). GO analyses were performed using PANTHER GO Enrichment Analysis (The Gene Ontology Consortium, 2000, 2019; Mi et al., 2019) (http://geneontology.org) with the PANTHER Overrepresentation Test (Released 20210224). Genes were annotated using the GO database (Released 2021-02-01) and categorized by Biological Processes. Venn diagrams were drawn with BioVenn (Hulsen et al., 2008) (http://www.biovenn.nl) and processed with Inkscape version 0.92.4 (https://inkscape.org). Heat maps were drawn using the Color Scale tool in Microsoft Excel with the expression data of the splice variant 1 for each gene.

### RT–qPCR

Extracted total RNA was subjected to first-strand cDNA synthesis using a PrimeScript RT reagent kit (Takara, Kusatsu, Japan). Quantitative real-time PCR was performed with Thunderbird SYBR qPCR mix (Toyobo, Osaka, Japan) in a Stratagene MX3000P real-time qPCR system (Agilent Technologies, Santa Clara, CA, USA). The PCR program consisted of a 1-min denaturation step at 95°C, followed by 40 cycles of 15 s at 95°C and 60 s at 60°C. The transcript levels were calculated using the standard curve method and normalized to that of the internal control *PROTEIN PHOSPHATASE 2A-3* (*PP2A3*) (Rymen et al., 2017). The primer sequences are listed in Supplemental Table S2. The primer sequences for *PP2A3* are from Rymen et al. (2017), those for *YUC1* are from Sugawara et al. (2015), and those for *PLT3* and *PLT5* are from Ohbayashi et al. (2022).

## Supplementary Material

koac218_Supplementary_Data

## Data Availability

The RNA-seq data are deposited in the National Center for Biotechnology Information (NCBI) Gene Expression Omnibus with accession numbers GSE178645 and GSE204767. All data needed to evaluate the conclusions in the article are present in the article and/or the Supplemental Information. Sequence data from this article can be found in the Arabidopsis Genome Initiative under the following accession numbers: *hag1-1* (SALK_150784), *hag1-2* (SALK_030913), *hag2* (SALK_051832), *hag3-1* (GABI_555H06), *hag3-2* (SALKseq_060819), *ham1* (SALK_027726), *ham2* (SALK_106046), *hac1* (SALK_080380), *hac2* (SALK_049434), *hac4* (SALK_045791), *hac5* (SAIL_49_D10), *hac12* (SALK_012469), *haf1-1* (SAIL_256_D10), *haf1-2* (SALK_110848), *haf2* (SALK_110029), *yuc1* (SALK_106293), *yuc4* (SM_3_16128), *cyp79B2* (SALK_130570C), *cyp79B3* (SALKseq_066556), *yuc3* (GABI_376G12), *yuc5* (CSHL_GT6160), *yuc7* (SALK_059832), *yuc8* (SM_3_23299) *yuc9* (SALK_762_D07), *myb3r1-1* (SALK_018482), *myb3r4-1* (SALK_059819), *arf7-1* (SALK_040394), *arf19-4* (SALK_009879), *lbd16-1* (SALK_095791), *lbd18-1* (SALK_038125), and *lbd33-1* (SAIL_95_H10).

## Supplemental data

The following materials are available in the online version of this article.

**Supplemental Figure S1.** Cell cycle reinitiation and callus formation described with a newly established culture system and time-lapse confocal microscopy.

**Supplemental Figure S2.** Transcriptional changes of chloroplast-related genes during protoplast reprograming.

**Supplemental Figure S3.** Time-lapse confocal microscopy images of leaf mesophyll protoplasts that did or did not undergo cell division.

**Supplemental Figure S4.** Roles of HATs in protoplast cell cycle reinitiation.

**Supplemental Figure S5.** Roles of auxin biosynthesis in protoplast cell cycle reinitiation.

**Supplemental Figure S6.** Association between auxin response and the morphological changes during reprograming of protoplasts.

**Supplemental Figure S7.** Roles of histone acetylation, auxin biosynthesis, and exogenous 2,4-D in regulating expression of cell cycle genes during protoplast reprograming.

**Supplemental Figure S8.** Roles of auxin signaling components in protoplast cell cycle reinitiation.

**Supplemental Figure S9.** Roles of ARF7 and ARF19 in regulating expression of cell cycle genes during protoplast reprograming.

**Supplemental Table S1.** Composition of the media and reagents used in this study.

**Supplemental Table S2.** Primers used in this study.

**Supplemental Data Set 1.** Expression data for chloroplast-related genes.

**Supplemental Data Set 2.** Expression data for the genes encoding enzymes for cell wall biosynthesis.

**Supplemental Data Set 3.** Expression data for the genes encoding HATs.

**Supplemental Data Set 4.** Lists of the DEGs shown in Figure 2C.

**Supplemental Data Set 5.** Expression data for the genes encoding TCA cycle and respiratory chain components.

**Supplemental Data Set 6.** A list of genes involved in biosynthesis and metabolism of indole-containing compounds that show significantly stronger upregulation in the control condition compared to that in the MB-3 condition.

**Supplemental Data Set 7.** Expression data for IAA biosynthesis-related genes.

**Supplemental Data Set 8.** Expression data for cell cycle genes during the first week of protoplast culture.

**Supplemental Data Set 9.** Expression data for auxin-related genes.

**Supplemental Data Set 10.** Expression data for cell cycle genes during the first week of protoplast culture in the WT and *nph4-1 arf19-1* protoplasts.

## Notes

Y.S. and K.S. conceived the project. Y.S. and K.S. designed the experiments, and Y.S. conducted most of genetic and cell biological analyses except for RNA sequencing and mapping which were performed by T.S. A.K. generated *pHAG1:HAG1-GFP/hag1-1* plants. S.P. and L.D.V. generated *pCAB3:H2A-eGFP* plants. S.S. and M.M. provided *pVHP1:VHP1-mGFP* seeds. Y.S. and K.S. wrote the manuscript with help from the co-authors.

The author responsible for distribution of materials integral to the findings presented in this article in accordance with the policy described in the Instructions for Authors (https://academic.oup.com/plcell) is: Keiko Sugimoto (keiko.sugimoto@riken.jp).

## References

[koac218-B1] Andriankaja M , Dhondt S, De Bodt S, Vanhaeren H, Coppens F, De Milde L, Mühlenbock P, Skirycz A, Gonzalez N, Beemster GTS, et al (2012) Exit from proliferation during leaf development in *Arabidopsis thaliana*: a not-so-gradual process. Dev Cell 22: 64–78 22227310 10.1016/j.devcel.2011.11.011

[koac218-B2] Bacete L , Mélida H, Miedes E, Molina A (2018) Plant cell wall-mediated immunity: cell wall changes trigger disease resistance responses. Plant J 93: 614–636 29266460 10.1111/tpj.13807

[koac218-B3] Balasubramanyam K , Altaf M, Varier RA, Swaminathan V, Ravindran A, Sadhale PP, Kundu TK (2004) Polyisoprenylated benzophenone, garcinol, a natural histone acetyltransferase inhibitor, represses chromatin transcription and alters global gene expression. J Biol Chem 279: 33716–33726 15155757 10.1074/jbc.M402839200

[koac218-B4] Beemster GTS , De Veylder L, Vercruysse S, West G, Rombaut D, Van Hummelen P, Galichet A, Gruissem W, Inzé D, Vuylsteke M (2005) Genome-wide analysis of gene expression profiles associated with cell cycle transitions in growing organs of Arabidopsis. Plant Physiol 138: 734–743 15863702 10.1104/pp.104.053884 PMC1150392

[koac218-B5] Benková E , Michniewicz M, Sauer M, Teichmann T, Seifertová D, Jürgens G, Friml J (2003) Local, efflux-dependent auxin gradients as a common module for plant organ formation. Cell 115: 591–602 14651850 10.1016/s0092-8674(03)00924-3

[koac218-B6] Biel M , Kretsovali A, Karatzali E, Papamatheakis J, Giannis A (2004) Design, synthesis, and biological evaluation of a small-molecule inhibitor of the histone acetyltransferase Gcn5. Angew Chem Int Ed 43: 3947–3976 10.1002/anie.200453879 15274229

[koac218-B7] Bowers EM , Yan G, Mukherjee C, Orry A, Wang L, Holbert MA, Crump NT, Hazzalin CA, Liszczak G, Yuan H, et al (2010) Virtual ligand screening of the p300/CBP histone acetyltransferase: identification of a selective small molecule inhibitor. Chem Biol 17: 471–482 20534345 10.1016/j.chembiol.2010.03.006 PMC2884008

[koac218-B8] Candeale J , Demuynck K, Mosoti D, Beemster GTS, Inzé D, Nelissen H (2014) Differential methylation during maize leaf growth targets developmentally regulated genes. Plant Physiol 164: 1350–1364 24488968 10.1104/pp.113.233312 PMC3938625

[koac218-B9] Carle SA , Bates GW, Shannon TA (1998) Hormonal control of gene expression during reactivation of the cell cycle in tobacco mesophyll protoplasts. J Plant Growth Regul 17: 221–230 9892745 10.1007/pl00007038

[koac218-B10] Chen L , Tong J, Xiao L, Ruan Y, Liu J, Zeng M, Huang H, Wang JW, Xu L (2016) *YUCCA*-mediated auxin biogenesis is required for cell fate transition occurring during de novo root organogenesis in Arabidopsis. J Exp Bot 67: 4273–4284 27255928 10.1093/jxb/erw213 PMC5301932

[koac218-B11] Chen P , Takatsuka H, Takahashi N, Kurata R, Fukao Y, Kobayashi K, Ito M, Umeda M (2017) *Arabidopsis* R1R2R3-Myb proteins are essential for inhibiting cell division in response to DNA damage. Nat Commun 8: 1–12 28935922 10.1038/s41467-017-00676-4 PMC5608833

[koac218-B12] Chen Q , Dai X, De-Paoli H, Cheng Y, Takebayashi Y, Kasahara H, Kamiya Y, Zhao Y (2014) Auxin overproduction in shoots cannot rescue auxin deficiencies in Arabidopsis roots. Plant Cell Physiol 55: 1072–1079 24562917 10.1093/pcp/pcu039 PMC4051135

[koac218-B13] Christianson ML , Warnick DA (1983) Competence and determination in the process of *in vitro* shoot organogenesis. Dev Biol 95: 288–293 6825936 10.1016/0012-1606(83)90029-5

[koac218-B14] Chupeau MC , Granier F, Pichon O, Renou JP, Gaudin V, Chupeau Y (2013) Characterization of the early events leading to totipotency in an Arabidopsis protoplast liquid culture by temporal transcript profiling. Plant Cell 25: 2444–2463 23903317 10.1105/tpc.113.109538 PMC3753376

[koac218-B15] Chupeau MC , Lemoine M, Chupeau Y (1993) Requirement of thidiazuron for healthy protoplast development to efficient tree regeneration of a hybrid poplar (*Populus tremula* β p. alba). J Plant Physiol 141: 601–609

[koac218-B16] Clough SJ , Bent AF (1998) Floral dip: a simplified method for *Agrobacterium*-mediated transformation of *Arabidopsis thaliana*. Plant J 16: 735–743 10069079 10.1046/j.1365-313x.1998.00343.x

[koac218-B17] Dai X , Mashiguchi K, Chen Q, Kasahara H, Kamiya Y, Ojha S, DuBois J, Ballou D, Zhao Y (2013) The biochemical mechanism of auxin biosynthesis by an Arabidopsis YUCCA flavin-containing monooxygenase. J Biol Chem 288: 1448–1457 23188833 10.1074/jbc.M112.424077 PMC3548458

[koac218-B18] Damm B , Willmitzer L (1988) Regeneration of fertile plants form protoplasts of different *Arabidopsis thaliana* genotypes. Mol Gen Genet 213: 15–20

[koac218-B19] Delbarre A , Muller P, Inhoff V, Guern VJ (1996) Comparison of mechanisms controlling uptake and accumulation of 2,4-dichlorophenoxy acetic acid, naphthalene-1-acetic acid, and indole-3-acetic acid in suspension-cultured tobacco cells. Planta 198: 532–541 28321663 10.1007/BF00262639

[koac218-B20] Dello Ioio R , Nakamura K, Moubayidin L, Perilli S, Taniguchi M, Morita MT, Aoyama T, Costantino P, Sabatini S (2008) A genetic framework for the control of cell division and differentiation in the root meristem. Science 322: 1380–1384 19039136 10.1126/science.1164147

[koac218-B21] De Marco A , Roubelakis-Angelakis KA (1996) The complexity of enzymic control of hydrogen peroxide concentration may affect the regeneration potential of plant protoplasts. Plant Physiol 110: 137–145 12226176 10.1104/pp.110.1.137 PMC157702

[koac218-B22] Dovzhenko A , Bergen U, Koop HU (1998) Thin-alginate-layer technique for protoplast culture of tobacco leaf protoplasts: shoot formation in less than two weeks. Protoplasma 204: 114–118

[koac218-B23] Eyer L , Vain T, Pařízková B, Oklestkova J, Barbez E, Kozubíková H, Oispíšil T, Wierzbicka R, Kleine-Vehn J, Fránek M, et al (2016) 2,4-D and IAA amino acid conjugates show distinct metabolism in *Arabidopsis*. PLoS One 11: e0159269 27434212 10.1371/journal.pone.0159269 PMC4951038

[koac218-B24] Fan M , Xu C, Xu K, Hu Y (2012) LATERAL ORGAN BOUNDERIES DOMAIN transcription factors direct callus formation in *Arabidopsis* regeneration. Cell Res 22: 1169–1180 22508267 10.1038/cr.2012.63 PMC3391013

[koac218-B25] Favero DS , Kawamura A, Shibata M, Takebayashi A, Jung JH, Suzuki T, Jaeger KE, Ishida T, Iwase A, Wigge PA, et al (2020) AT-hook transcription factors restrict petiole growth by antagonizing PIFs. Curr Biol 30: 1454–1466 32197081 10.1016/j.cub.2020.02.017

[koac218-B26] Fendrych M , Van Hautegem T, Van Durme M, Olvera-Carrillo Y, Huysmans M, Karimi M, Lippens S, Guérin CJ, Krebs M, Schumacher K, et al (2014) Programmed cell death controlled by ANAC033/SOMBRERO determines root cap organ size in *Arabidopsis*. Curr Biol 24: 931–940 24726156 10.1016/j.cub.2014.03.025

[koac218-B27] Fukaki H , Tameda S, Masuda H, Tasaka M (2002) Lateral root formation is blocked by g gain-of-function mutation in the *SOLITARY-ROOT/IAA14* gene of *Arabidopsis*. Plant J 29: 153–168 11862947 10.1046/j.0960-7412.2001.01201.x

[koac218-B28] Goh T , Joi S, Mimura T, Fukaki H (2012a) The establishment of asymmetry in *Arabidopsis* lateral root founder cells is regulated by LBD16/ASL18 and related LBD/ASL proteins. Development 139: 883–893 22278921 10.1242/dev.071928

[koac218-B29] Goh T , Kasahara H, Mimura T, Kamiya Y, Fukaki H (2012b) Multiple AUX/IAA-ARF modules regulate lateral root formation: the role of *Arabidopsis* SHY2/IAA3-mediated auxin signalling. Philos Trans R Soc B 367: 1461–1468 10.1098/rstb.2011.0232 PMC3321683 22527388

[koac218-B30] Haga N , Kato K, Murase M, Araki S, Kubo M, Demura T, Suzuki K, Müller I, Voß U, Jürgens G, et al (2007) R1R2R3-Myb proteins positively regulate cytokinesis through activation of KNOLLE transcription in *Arabidopsis thaliana*. Development 134: 1101–1110 17287251 10.1242/dev.02801

[koac218-B31] Haga N , Kobayashi K, Suzuki T, Maeno K, Kubo M, Ohtani M, Mitsuda N, Demura T, Nakamura K, Jürgens G, et al (2011) Mutations in *MYB3R*1 and *MYB3R4* cause pleiotropic developmental defects and preferential down-regulation of multiple G2/M-specific genes in Arabidopsis. Plant Physiol 157: 706–717 21862669 10.1104/pp.111.180836 PMC3192584

[koac218-B32] Hall RD , Verhoeven HA, Krens FA (1995) Computer-assisted identification of protoplasts responsible for rare division events reveals guard-cell totipotency. Plant Physiol 107: 1379–1386 12228442 10.1104/pp.107.4.1379 PMC157273

[koac218-B33] He W , Brumos J, Li H, Ji Y, Ke M, Gong X, Zeng Q, Li W, Zhang X, An F, et al (2011) A small-molecule screen identifies L-kynurenine as a competitive inhibitor of TAA1/TAR activity in ethylene-directed auxin biosynthesis and root growth in *Arabidopsis*. Plant Cell 23: 3944–3960 22108404 10.1105/tpc.111.089029 PMC3246337

[koac218-B34] Hulsen T , De Vlieg J, Alkema JW (2008) BioVenn - a web application for the comparison and visualization of biological lists using area-proportional Venn diagrams. BMC Genomics 9: 1–6 18925949 10.1186/1471-2164-9-488 PMC2584113

[koac218-B35] Ikeuchi M , Favero DS, Sakamoto Y, Iwase A, Coleman D, Rymen B, Sugimoto K (2019) Molecular mechanisms of plant regeneration. Annu Rev Plant Biol 70: 377–406 30786238 10.1146/annurev-arplant-050718-100434

[koac218-B36] Ikeuchi M , Iwase A, Sugimoto K (2015) Control of plant cell differentiation by histone modification and DNA methylation. Curr Opin Plant Biol 28: 60–67 26454697 10.1016/j.pbi.2015.09.004

[koac218-B37] Iwase A , Mitsuda N, Koyama T, Hiratsu K, Kojima M, Arai T, Inoue Y, Seki M, Sakakibara H, Sugimoto K, et al (2011) The AP2/ERF transcription factor WIND1 control cell dedifferentiation in Arabidopsis. Curr Biol 21: 508–514 21396822 10.1016/j.cub.2011.02.020

[koac218-B38] Kalve S , De Vos D, Beemster GTS (2014) Leaf development: a cellular perspective. Front Plant Sci 5: 1–25 10.3389/fpls.2014.00362 PMC4116805 25132838

[koac218-B39] Kao KN , Keller WA, Miller RA (1970) Cell division in newly formed cells from protoplasts of soybean. Exp Cell Res 62: 338–340 5531376 10.1016/0014-4827(70)90563-x

[koac218-B40] Kim JY , Yang W, Forner J, Lohmann JU, Noh B, Noh YS (2018) Epigenetic reprogramming by histone acetyltransferase HAG1/AtGCN5 is required for pluripotency acquisition in *Arabidopsis*. EMBO J 37: e98726 30061313 10.15252/embj.201798726 PMC6187204

[koac218-B41] Kobayashi K , Suzuki T, Iwata E, Nakamichi N, Suzuki T, Chen P, Ohtani M, Ishida T, Hosoya H, Müller S, et al (2015) Transcriptional repression by MYB3R proteins regulates plant organ growth. EMBO J 34: 1992–2007 26069325 10.15252/embj.201490899 PMC4551348

[koac218-B42] Kondo Y , Fujita T, Sugiyama M, Fukuda H (2015) A novel system for xylem cell differentiation in *Arabidopsis thaliana*. Mol Plant 8: 612–621 25624147 10.1016/j.molp.2014.10.008

[koac218-B43] Kuki H , Higaki T, Yokoyama R, Kuroha T, Shinohara N, Hasezawa S, Nishitani K (2017) Quantitative confocal imaging method for analyzing cellulose dynamics during cell wall regeneration in Arabidopsis mesophyll protoplasts. Plant Direct 1: 1–10 10.1002/pld3.21 PMC6508514 31245675

[koac218-B44] Langmead B , Salzberg SL (2012) Fast gapped-read alignment with Bowtie 2. Nat Methods 9: 357–359 22388286 10.1038/nmeth.1923 PMC3322381

[koac218-B45] Lee K , Park OS, Seo PJ (2017) *Arabidopsis* ATXR2 deposits H3K36me3 at the promoters of *LBD* genes to facilitate cellular dedifferentiation. Sci Signal 10: eaan0316 29184030 10.1126/scisignal.aan0316

[koac218-B46] Martínez-Reyes I , Chandel NS (2020) Mitochondrial TCA cycle metabolites control physiology and disease. Nat Commun 11: 102 31900386 10.1038/s41467-019-13668-3 PMC6941980

[koac218-B47] Masson J , Paszkowski J (1992) The culture response of *Arabidopsis thaliana* protoplasts is determined by the growth conditions of donor plants. Plant J 2: 829–833

[koac218-B48] Matosevich R , Cohen I, Gil-Yarom N, Modrego A, Friedlander-Shani L, Verna C, Scarpella E, Efroni I (2020) Local auxin biosynthesis is required for root regeneration after wounding. Nat. Plants 6: 1020–1030 32747761 10.1038/s41477-020-0737-9

[koac218-B49] Mi H , Muruganujan A, Ebert D, Huang X, Thomas PD (2019) PANTHER version 14: more genomes, a new PANTHER GO-slim and improvements in enrichment analysis tools. Nucleic Acid Res 47: D419–D426 30407594 10.1093/nar/gky1038 PMC6323939

[koac218-B50] Mielke S , Gasperini D (2019) Interplay between plant cell walls and jasmonate production. Plant Cell Physiol 49: 2629–2637 10.1093/pcp/pcz119 31241137

[koac218-B51] Nagpal P , Walker LM, Young JC, Sonawala A, Timple C, Estelle M, Reed JW (2000) *AXR2* encodes a member of the Aux/IAA protein family. Plant Physiol 123: 563–573 10859186 10.1104/pp.123.2.563 PMC59024

[koac218-B52] Nishimura T , Hayashi K, Suzuki H, Gyohda A, Takaoka C, Sakaguchi Y, Matsumoto S, Kasahara H, Sakai T, Kato JI, et al (2014) Yucasin is a potent inhibitor of YUCCA, a key enzyme in auxin biosynthesis. Plant J 77: 352–366 24299123 10.1111/tpj.12399

[koac218-B53] Ohbayashi I , Sakamoto Y, Kuwae H, Kasahara H, Sugiyama M (2022) Enhancement of shoot regeneration by treatment with inhibitors of auxin biosynthesis and transport during callus induction in tissue culture of *Arabidopsis thaliana*. Plant Biotechnol 39: 43–50 10.5511/plantbiotechnology.21.1225a PMC9200084 35800968

[koac218-B54] Okushima Y , Overvoorde PJ, Arima K, Alonso JM, Chan A, Chang C, Ecker JR, Hughes B, Lui A, Nguyen D, et al (2005) Functional genomic analysis of the *AUXIN RESPONSE FACTOR* gene family members in Arabidopsis thaliana: unique and overlapping functions of *ARF7* and *ARF19*. Plant Cell 17: 444–463 15659631 10.1105/tpc.104.028316 PMC548818

[koac218-B55] Pandey S , Wang XQ, Coursol SA, Assmann SM (2002) Preparation and applications of *Arabidopsis thaliana* guard cell protoplasts. New Phytol 153: 517–526 33863229 10.1046/j.0028-646X.2001.00329.x

[koac218-B56] Pasternak T , Miskolxzi P, Ayaydin F, Mészáros T, Dudits D, Fehér A (2000) Exogenous auxin and cytokinin dependent activation of CDKs and cell division in leaf protoplast-derived cell of alfalfa. Plant Growth Regul 32: 129–141

[koac218-B57] Pasternak TP , Prinsen E, Ayaydin F, Miskolczi P, Potters G, Asard H, Van Onckelen HA, Dudits D, Fehér A (2002) The role of auxin, pH, and stress in the activation of embryogenic cell division in leaf protoplast-derived cells of alfalfa. Plant Physiol 129: 1807–1819 12177494 10.1104/pp.000810 PMC166769

[koac218-B58] Prasad K , Grigg SP, Barkoulas M, Yadav RK, Sanchez-Perez GF, Pinon V, Blilou I, Hofhuis H, Dhonukshe P, Galinha C, et al (2011) *Arabidopsis* PLETHORA transcription factors conntrol phyllotaxis. Curr Biol 21: 1123–1128 21700457 10.1016/j.cub.2011.05.009

[koac218-B59] Piya S , Shrestha SK, Binder B, Stewart CN Jr, Hewezi T (2014) Protein-protein interaction and gene co-expression maps of ARFs and Aux/IAAs in Arabidopsis. Front Plant Sci 5: 1–9 10.3389/fpls.2014.00744 PMC4274898 25566309

[koac218-B60] Radhakrishnan D , Shanmukhan AP, Kareem A, Aiyaz M, Varapparambathu V, Toms A, Karstens M, Valsakumar D, Landge AN, Shaji A, et al (2020) A coherent feed-forward loop drives vascular regeneration in damaged aerial organs of plants growing in a nonrmal developmental context. Development 147: dev185710 32108025 10.1242/dev.185710

[koac218-B61] Ranocha P , Dima O, Nagt R, Felten J, Corratgé-Failline C, Novák O, Morreel K, Lacombe B, Martinez Y, Pfrunder S, et al (2013) Arabidopsis WAT1 is a vacuolar auxin transport facilitator required for auxin homoeostasis. Nat Commun 4: 1–9 10.1038/ncomms3625 PMC3826630 24129639

[koac218-B62] Robinson MD , McCarthy DJ, Smyth GK (2010) edgeR: a bioconductor package for differential expression analysis of digital gene expression data. Bioinformatics 26: 139–140 19910308 10.1093/bioinformatics/btp616 PMC2796818

[koac218-B63] Rymen B , Kawamura A, Lambolez A, Inagaki S, Takebayashi A, Iwase A, Sakamoto Y, Sako K, Favero DS, Ikeuchi M, et al (2019) Histone acetylation orchestrates wound-induced transcriptional activation and cellular reprogramming in Arabidopsis. Commun Biol 2: 1–15 31701032 10.1038/s42003-019-0646-5 PMC6828771

[koac218-B64] Rymen B , Kawamura A, Schäfer S, Breuer C, Iwase A, Shibata M, Ikeda M, Mitsuda N, Koncz C, Ohme-Takagi M, et al (2017) ABA suppresses root hair growth via the OBP4 transcriptional regulator. Plant Physiol 173: 1750–1762 28167701 10.1104/pp.16.01945 PMC5338652

[koac218-B65] Schilde-Rentschler L (1977) Role of the cell wall in the ability of tobacco protoplasts to form callus. Planta 135: 177–181 24420021 10.1007/BF00387168

[koac218-B66] Segami S , Makino S, Miyake A, Asaoka M, Maeshima M (2014) Dynamics of vacuoles and H^+^-pyrophosphatase visualized by monomeric green fluorescent protein in *Arabidopsis*: artifactual bulbs and native intravacuolar spherical structures. Plant Cell 26: 3416–3434 25118245 10.1105/tpc.114.127571 PMC4371836

[koac218-B67] Shahbazian MD , Grunstein M (2007) Functions of site-specific histone acetylation and deacetylation. Annu Rev Biochem 76: 75–100 17362198 10.1146/annurev.biochem.76.052705.162114

[koac218-B68] Sheahan MB , Rose RJ, McCurdy DW (2007) Actin-filament-dependent remodeling of the vacuole in cultured mesophyll protoplasts. Protoplasma 230: 141–152 17458629 10.1007/s00709-006-0236-5

[koac218-B69] Sugawara S , Mashiguchi K, Tanaka K, Hishiyama S, Sakai T, Hanada K, Kinoshita-Tsujimura K, Yu H, Dai X, Takebayashi Y, et al (2015) Distinct characteristics of indole-3-acetic acid and phenylacetic acid, two common auxins in plants. Plant Cell Physiol 56: 1641–1654 26076971 10.1093/pcp/pcv088 PMC4523386

[koac218-B70] Susek RE , Ausubel FM, Chory J (1993) Signal transduction mutants of Arabidopsis uncouple nuclear *CAB* and *RBCS* gene expression from chloroplast development. Cell 74: 787–799 7690685 10.1016/0092-8674(93)90459-4

[koac218-B71] Takebe I , Labib G, Melchers G (1971) Regeneration of whole plants from isolated mesophyll protoplasts of tobacco. Naturwissenschaften 58: 318–320

[koac218-B72] The Gene Ontology Consortium (2019) The gene ontology resource: 20 years and still GOing strong. Nucleic Acid Res. 47: D330–D338 30395331 10.1093/nar/gky1055 PMC6323945

[koac218-B73] The Gene Ontology Consortium (2000) Gene ontology: tool for the unification of biology. Nat Gen 25: 25–29 10.1038/75556 PMC3037419 10802651

[koac218-B74] Thomas MR , Rose RJ (1983) Plastid number and plastid structural changes associated with tobacco mesophyll protoplast culture and plant regeneration. Planta 158: 329–338 24264753 10.1007/BF00397335

[koac218-B75] Tiew TWY , Sheahan MB, Rose RJ (2015) Peroxisomes contribute to reactive oxygen species homeostasis and cell division induction in *Arabidopsis* protoplasts. Front Plant Sci 6: 1–16 26379686 10.3389/fpls.2015.00658 PMC4549554

[koac218-B76] Uehara T , Okushima Y, Mimura T, Tasaka M, Fukaki H (2008) Domain II mutations in CRANE/IAA18 suppress lateral root formation and affect shoot development in *Arabidopsis thaliana*. Plant Cell Physiol 49: 1025–1038 18505759 10.1093/pcp/pcn079

[koac218-B77] Ulmasov T , Murfett J, Hagen G, Guilfoyle TJ (1997) Aux/IAA proteins repress expression of reporter genes containing natural and highly active synthetic auxin response elements. Plant Cell 9: 1963–1971 9401121 10.1105/tpc.9.11.1963 PMC157050

[koac218-B78] Wang S , Tiwari SB, Hagen G, Guilfoyle GTJ (2005) *AUXIN RESPONSE FACTOR7* restores the expression of auxin-responsive genes in mutant Arabidopsis leaf mesophyll protoplasts. Plant Cell 17: 1979–1993 15923351 10.1105/tpc.105.031096 PMC1167546

[koac218-B79] Williams L , Zhao J, Morozova N, Li Y, Avivi Y, Grafi G (2003) Chromatin reorganization accompanying cellular dedifferentiation is associated with modifications of histone H3, redistribution od HP1, and activation of E2F-target genes. Dev Dyn 228: 113–120 12950085 10.1002/dvdy.10348

[koac218-B80] Yang W , Cortijo S, Korsbo N, Roszak P, Schiessl K, Gurzadyan A, Wightman R, Jönsson H, Meyerowitz E (2021) Molecular mechanism of cytokinin-activated cell division in Arabidopsis. Science 10.1126/science.abe2305. 10.1126/science.abe2305 PMC8166333 33632892

[koac218-B81] Zhang TQ , Lian H, Zhou CM, Xu L, Jiao Y, Wang JW (2017) A two-step model for de novo activation of *WUSCHEL* during plant shoot regeneration. Plant Cell 29: 1073–1087 28389585 10.1105/tpc.16.00863 PMC5466026

[koac218-B82] Zhao J , Morozova N, Williams L, Libs L, Avivi Y, Grafi G (2001) Two phases of chromatin decondensation during dedifferentiation of plant cells. J Biol Chem 276: 22772–22778 11274191 10.1074/jbc.M101756200

[koac218-B83] Zhao Y , Hull AK, Gupta NR, Goss KA, Alonso J, Ecker JR, Normanly J, Chory J, Celenza JL (2002) Trp-dependent auxin biosynthesis in *Arabidopsis*: involvement of cytochrome P40s CYP79B2 and CYP79B3. Gen Dev 16: 3100–3112 10.1101/gad.1035402 PMC187496 12464638

