# Supplementary material for: Transcriptional activation of auxin biosynthesis drives developmental reprogramming of differentiated cells
Source: Plant Cell. 2022 Aug 4;34(11):4348–65. doi: 10.1093/plcell/koac218 (PMC9614439; doi:10.1093/plcell/koac218)
Supplement: koac218_Supplementary_Data [file koac218_supplementary_data.zip › koac218_Supplementary_Data/tpc.21.01024Supplemental Figures and Tables.pdf]

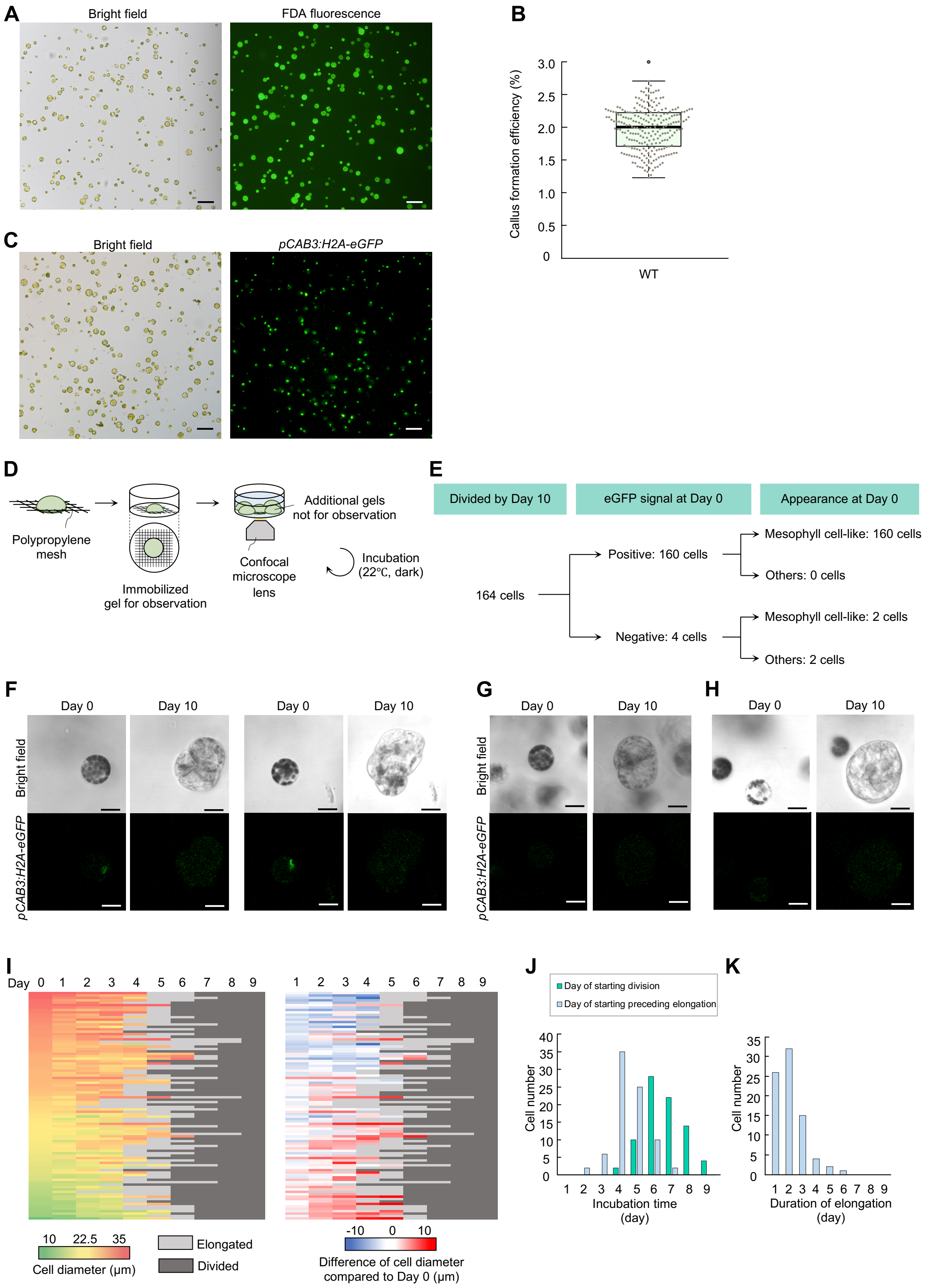
A
Bright field
FDA fluorescence
B
Callus formation efficiency (%)
3.0
2.5
2.0
1.5
1.0
0.5
0
WT
C
Bright field
pCAB3:H2A-eGFP
D
Polypropylene mesh
Immobilized gel for observation
Additional gels not for observation
Confocal microscope lens
Incubation (22℃, dark)
E
Divided by Day 10
eGFP signal at Day 0
Appearance at Day 0
164 cells
Positive: 160 cells
Negative: 4 cells
Mesophyll cell-like: 160 cells
Others: 0 cells
Mesophyll cell-like: 2 cells
Others: 2 cells
F
G
H
Day 0
Day 10
Bright field
pCAB3:H2A-eGFP
I
Day 0 1 2 3 4 5 6 7 8 9
10 22.5 35
Cell diameter (μm)
Elongated
Divided
-10 0 10
Difference of cell diameter compared to Day 0 (μm)
J
Day of starting division
Day of starting preceding elongation
Cell number
Incubation time (day)
K
Cell number
Duration of elongation (day)

**Supplemental Figure S1.**

**Cell cycle reinitiation and callus formation described with a newly established culture system and time-lapse confocal microscopy.** (**A**) Bright-field and fluorescence microscopy images of freshly isolated WT protoplasts. FDA staining indicates that 98.66 ± 0.19 % (standard error, n = 10) of freshly isolated protoplasts are viable. (**B**) Callus formation efficiency of WT protoplasts. Average efficiency is 1.98 ± 0.33%. n = 240 from 48 biological replicates. Each dot shows callus formation efficiency calculated from one gel. In the box plot, the median is represented by a black line and the upper and lower quartiles are represented by the upper and lower ends of the box respectively. (**C**) Bright-field and fluorescence microscopy images of protoplasts freshly isolated from plants carrying a mesophyll cell-specific marker *pCAB3:H2A-eGFP*. Quantitative analysis indicates that 94.63 ± 0.51% of freshly isolated protoplasts show distinct nuclear-localized H2A-eGFP expression (standard error, n = 15 from 2 biological replicates). (**D**) Diagram showing the time-lapse confocal microscopy procedure used to track individual protoplasts. (**E**) Classification of 164 protoplasts carrying *pCAB3:H2A-eGFP* that underwent cell division by Day 10 based on H2A-eGFP expression and appearance at Day 0. Among 164 cells that divided by Day 10, 160 cells were eGFP positive and had mesophyll cell-like appearance at Day 0. Among the 4 cells that were eGFP negative at Day 0, 2 cells had mesophyll cell-like appearance and the other 2 had guard cell-like appearance based on the density of chloroplasts. (**F**) Two examples of *pCAB3:H2A-eGFP* protoplasts that are H2A-eGFP-positive and mesophyll cell-like at Day 0. Both protoplasts divided by Day 10. (**G**) A *pCAB3:H2A-eGFP* protoplast that is H2A-eGFP-negative and mesophyll cell-like at Day 0. The protoplast divided by Day 10. (**H**) A *pCAB3:H2A-eGFP* protoplast that is H2A-eGFP-negative and guard cell-like at Day 0. The protoplasts divided by Day 10. (**I**) Heat maps representing cell size dynamics of protoplasts that reinitiate cell division between Day 4 and Day 9. Each row shows cell diameter (left panel) and change in cell diameter comparted to Day 0 (right panel) for individual protoplasts isolated from WT, *DR5rev:GFP* or *pVHP1:VHP1-mGFP* plants. Among 94 protoplasts tested in this experiment, 80 cells underwent cell elongation before cell division. (**J**) Timing of cell elongation and cell division for 80 cells from (I). (**K**) Duration of cell elongation (right panel) for 80 cells from (I). Scale bars are 100 µm (A), 100 µm (D) and 20 µm (F-H). **Supporting Figure 1.**

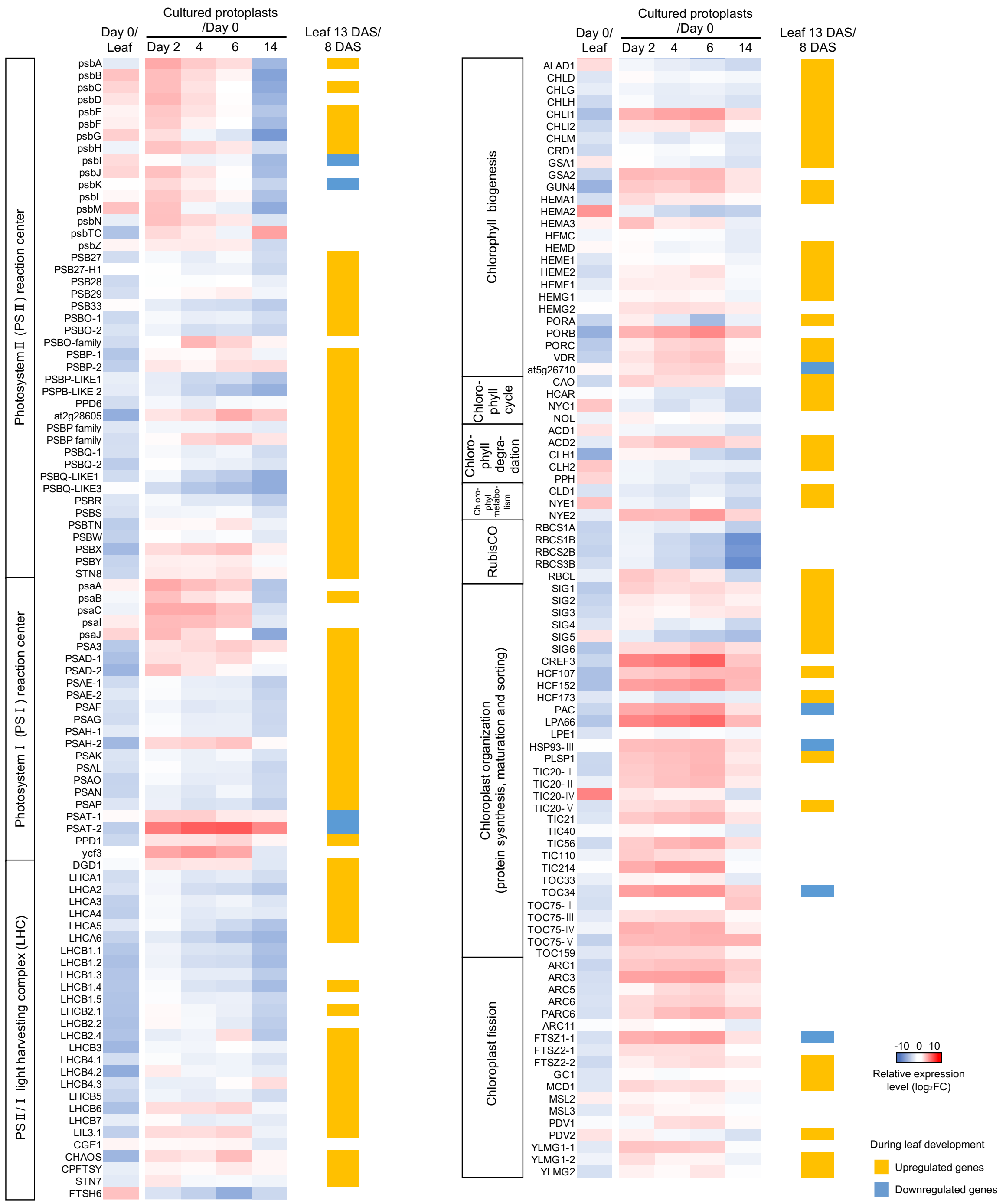


**Supplemental Figure S2.**

**Transcriptional changes of chloroplast-related genes during protoplast reprograming.** Heat map representing the transcriptional changes for genes implicated in chloroplast biogenesis and function. The leftmost column shows relative expression levels ($log_2FC$) in protoplasts at Day 0 compared to 23 DAS leaves to visualize the transcriptional changes during protoplast isolation. The second column shows relative expression levels (log2FC) in cultured protoplasts at Day 2, 4, 6 and 14 compared to Day 0 to visualize the transcriptional changes during subsequent protoplast culture. The right column shows genes significantly upregulated (orange) or downregulated (blue) during leaf development based on Andriankaja

et al. (2012). Gene sets were selected based on annotations at The Arabidopsis Information Resource (TAIR) (https://www.arabidopsis.org/index.jsp). **Supporting Figure 1.**

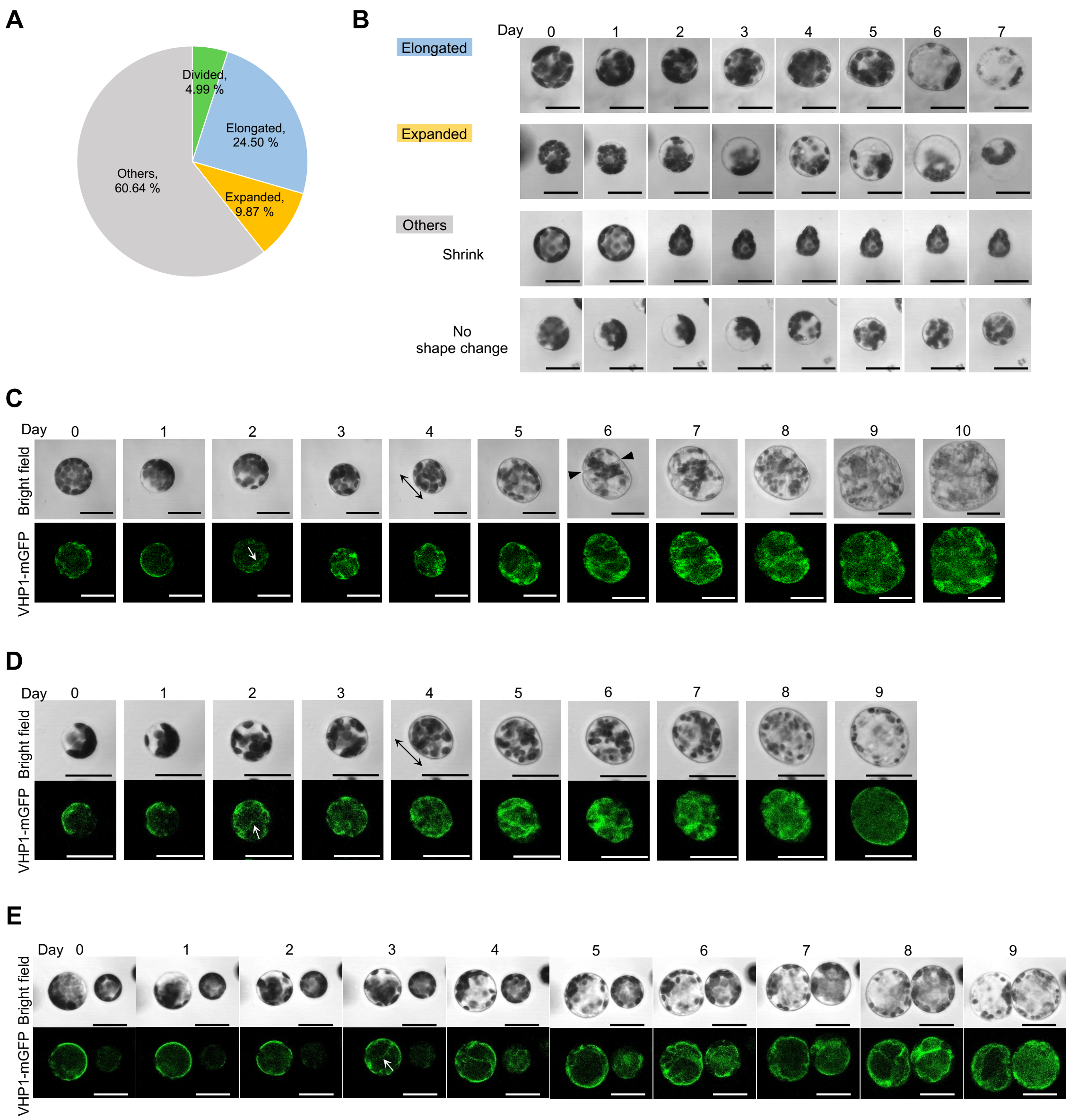


**Supplemental Figure S3.**

**Time-lapse confocal microscopy images of leaf mesophyll protoplasts that did or did not undergo cell division.** (**A**) Pie chart showing the percentages of protoplasts that underwent cell division (divided), those that elongated without cell division (elongated), those that expanded without cell division (expanded) and those that shrunk or displayed no shape changes (others) among 902 protoplasts isolated form *DR5rev:GFP* plants and used for time-lapse confocal microscopy. (**B**) Representative time-lapse images of elongated, expanded and other protoplasts from Day 0 to Day 7. (**C**) Another set of time-lapse images of a protoplast that underwent cell division. (**D**) Time-lapse images of a protoplast that elongated without cell division. (**E**) Time-lapse images of a protoplast that expanded without cell division. In (C) to (E), vacuolar morphology is visualized by VHP1-mGFP. The double-headed arrow indicates the direction of cell elongation and arrowheads mark the plane of initial cell division. The white arrow highlights the initial appearance of vacuolar strand-like structures. Scale bars are 30 μm (B-E). **Supporting Figure 1.**

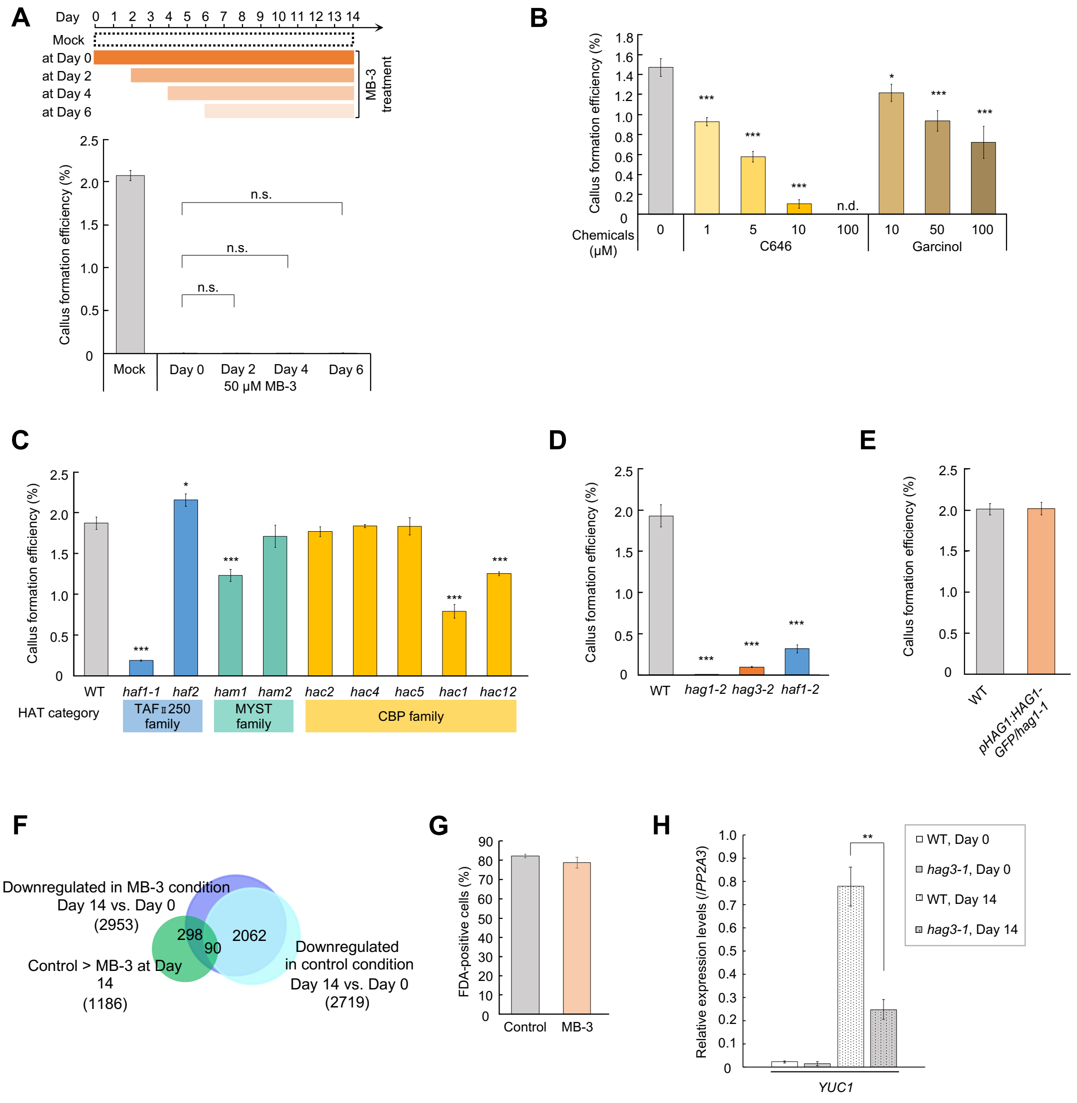


**Supplemental Figure S4.**

**Roles of histone acetyltransferases in protoplast cell cycle reinitiation.** (**A**) Callus formation efficiency of WT protoplasts treated with 50 μM MB-3 at different time points. Diagram shows the timing of MB-3 treatment. Data are represented as mean ± SEM. n = 15 from 3 biological replicates. n.s. not significant (two-tailed Welch's *t*-test compared to MB-3 treatment at Day 0). (**B**) Callus formation efficiency of WT protoplasts treated with C646 or garcinol. Data are represented as mean ± SEM. n = 30 from 6 biological replicates for the WT control and n = 15 from 3 biological replicates for all others. *$P < 0.05$, ***$P < 0.001$ (two-tailed Welch's *t*-test compared to WT control). n.d. not determined. (**C**) Callus formation efficiency of protoplasts isolated from WT and HAT mutants. Data are represented as mean ± SEM. n = 45 from 9 biological replicates for the WT and n = 10 or 15 from 2 or 3 biological replicates for mutants. *$P < 0.05$, *** $P < 0.001$ (two-tailed Welch's *t*-test compared to WT). (**D**) Callus formation efficiency of WT, *hag1-2*, *hag3-2* and *haf1-2* protoplasts. Data are represented as mean ± SEM. n = 25 from 5 biological replicates for the WT and n = 15 from 3 biological replicates for all others. *** $P < 0.001$ (two-tailed Welch's *t*-test compared to WT). (**E**) Callus formation efficiency of WT and *pHAG1:HAG1-GFP/hag1-1* protoplasts. Data are represented as mean ± SEM. n = 15 from 3 biological replicates. No statistical difference was detected (two-tailed Student's *t*-test compared to WT). (**F**) A Venn diagram of genes significantly downregulated in control and 50 μM MB-3 conditions. The green circle indicates genes

that show significantly higher expression in the control compared to the MB-3 condition at Day 14. Numbers in brackets show total numbers of genes included in each group. (**G**) The percentages of FDA-stained viable cells in WT protoplast populations cultured in control and 50 μM MB-3 conditions for 2 days. Data are represented as mean ± SEM (n = 10). No statistical difference was detected (two-tailed Welch's *t*-test). (**H**) The expression levels of *YUC1* in WT and *hag3-1* protoplasts cultured for 14 days. RT-qPCR data are represented as mean ± SEM (n = 3). The expression levels are normalized by those of the internal control *PP2A3*. $^{**}P < 0.01$ (two-tailed Student's *t*-test compared to WT). **Supporting Figure 2.**

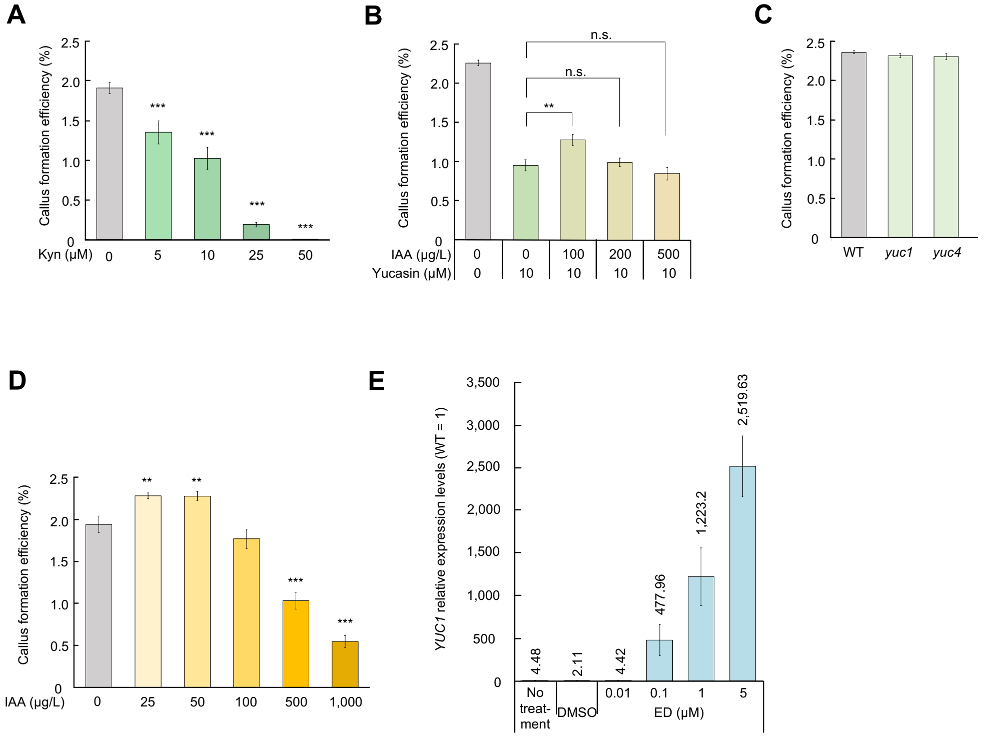


**Supplemental Figure S5.**

**Roles of auxin biosynthesis in protoplast cell cycle reinitiation.** (**A**) Callus formation efficiency of Kyn-treated WT protoplasts. Data are represented as mean ± SEM. n = 30 from 6 biological replicates for the WT control and n = 15 to 25 from 3 to 5 biological replicates. ***$P$ < 0.001 (two-tailed Welch's $t$-test compared to WT control). (**B**) Callus formation efficiency of Yucasin- and/or IAA-treated WT protoplasts. 0.4 µL of DMSO with or without Yucasin and 4 µL of EtOH with or without IAA were added to PCIM at the indicated final concentration at Day 0. Data are represented as mean ± SEM. n = 15 from 3 biological replicates. **$P$ < 0.01, n.s. not significant (two-tailed Student’s $t$-test compared to 10-µM Yucasin and 0-µM IAA treatment). (C) Callus formation efficiency of WT, *yuc1* and *yuc4* protoplasts. Data are represented as mean ± SEM. n = 15 from 3 biological replicates. No statistical difference was detected (two-tailed Student’s $t$-test compared to WT). (**D**) Callus formation efficiency of WT protoplasts incubated with PCIM supplemented with 0 to 1,000 µg/L IAA. 4 µL of EtOH with and without IAA was added to PCIM at the indicated final concentration at Day 0. Data are represented as mean ± SEM. n = 10 to15 from 2 to 3 biological replicates. **$P$ < 0.01, ***$P$ < 0.001 (two-tailed Welch's $t$-test compared to WT control). (**E**) β-estradiol-inducible expression of *YUC1* in *XVE-YUC1* plants. WT and *XVE-YUC1* seeds were sown in 3 mL of liquid half-strength MS medium and incubated at 22℃ under light with rotation on a Shake-LR (TAITEC) for 10 days. 3 µL of DMSO with or without ED was added to the MS medium at the indicated final concentration and plants were grown for another 24 hours. The expression levels of *YUC1* are normalized by those of the internal control *PP2A3* and shown as relative values compared to WT given the same treatments. The *YUC1* expression in WT remained constant throughout the treatments. Data are represented as mean ± SEM (n = 6). **Supporting Figure 3.**

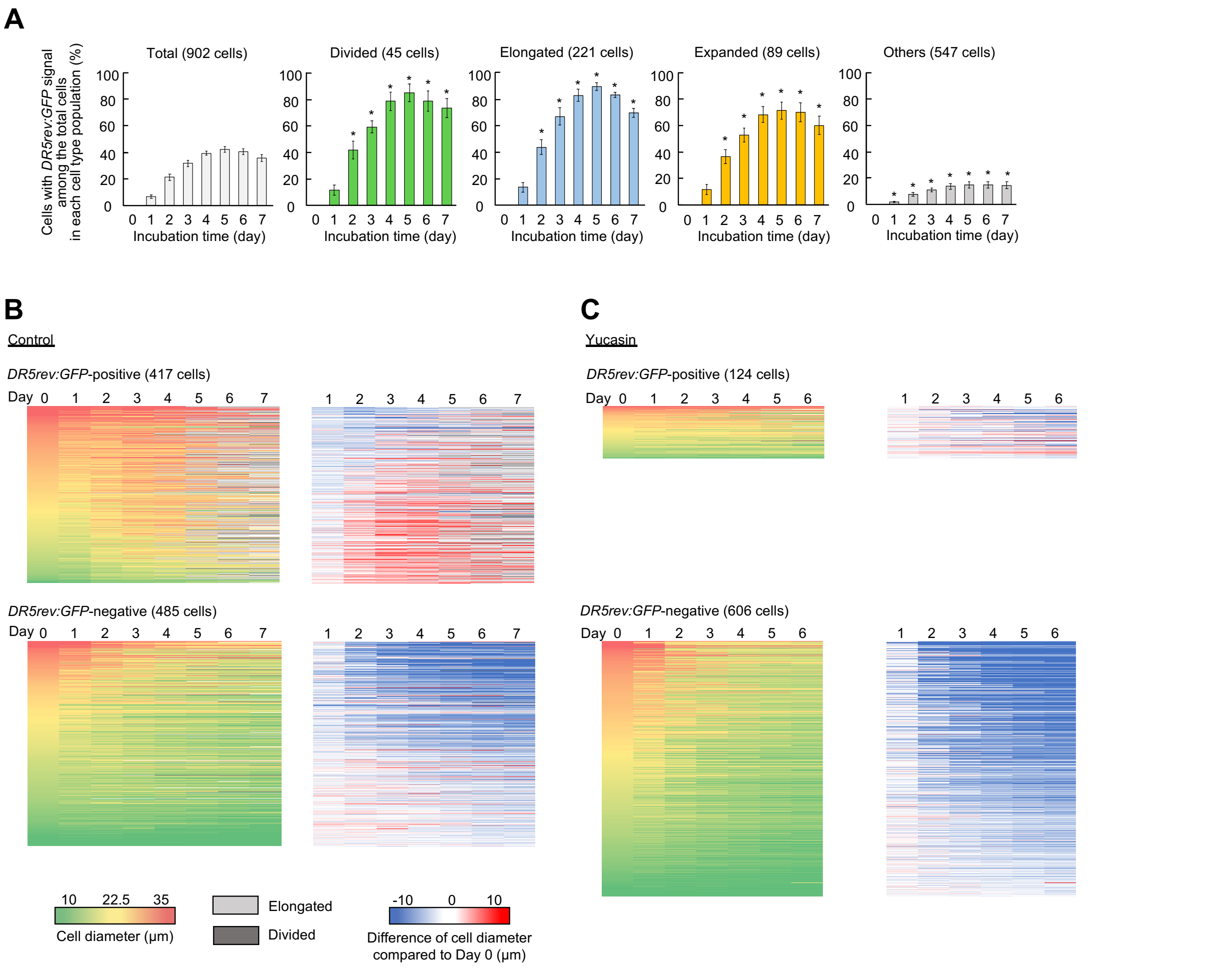


**Supplemental Figure S6.**

**Association between auxin response and the morphological changes during reprograming of protoplasts.** (**A**) Frequency of the *DR5rev:GFP*-expressing (positive) protoplasts among 902 total cells, 45 divided cells, 221 elongated cells, 89 expanded cells and 547 other cells. *DR5rev:GFP* protoplasts were incubated in the control condition. Data are represented as mean ± SEM. **P* < 0.05 (two-tailed Welch's *t*-test compared to 'Total' at the same time point). (**B**) Heat maps representing cell size dynamics of 417 *DR5rev:GFP*-positive and 485 *DR5rev:GFP*-negative protoplasts in the control condition. (**C**) Heat maps representing cell size dynamics of 124 *DR5rev:GFP*-positive and 606 *DR5rev:GFP*-negative protoplasts in the 50 µM yucasin condition. In (B) and (C), each row shows cell diameter (left panel) and change in cell diameter comparted to Day 0 (right panel) for individual *DR5rev:GFP* protoplasts. **Supporting Figure 4.**

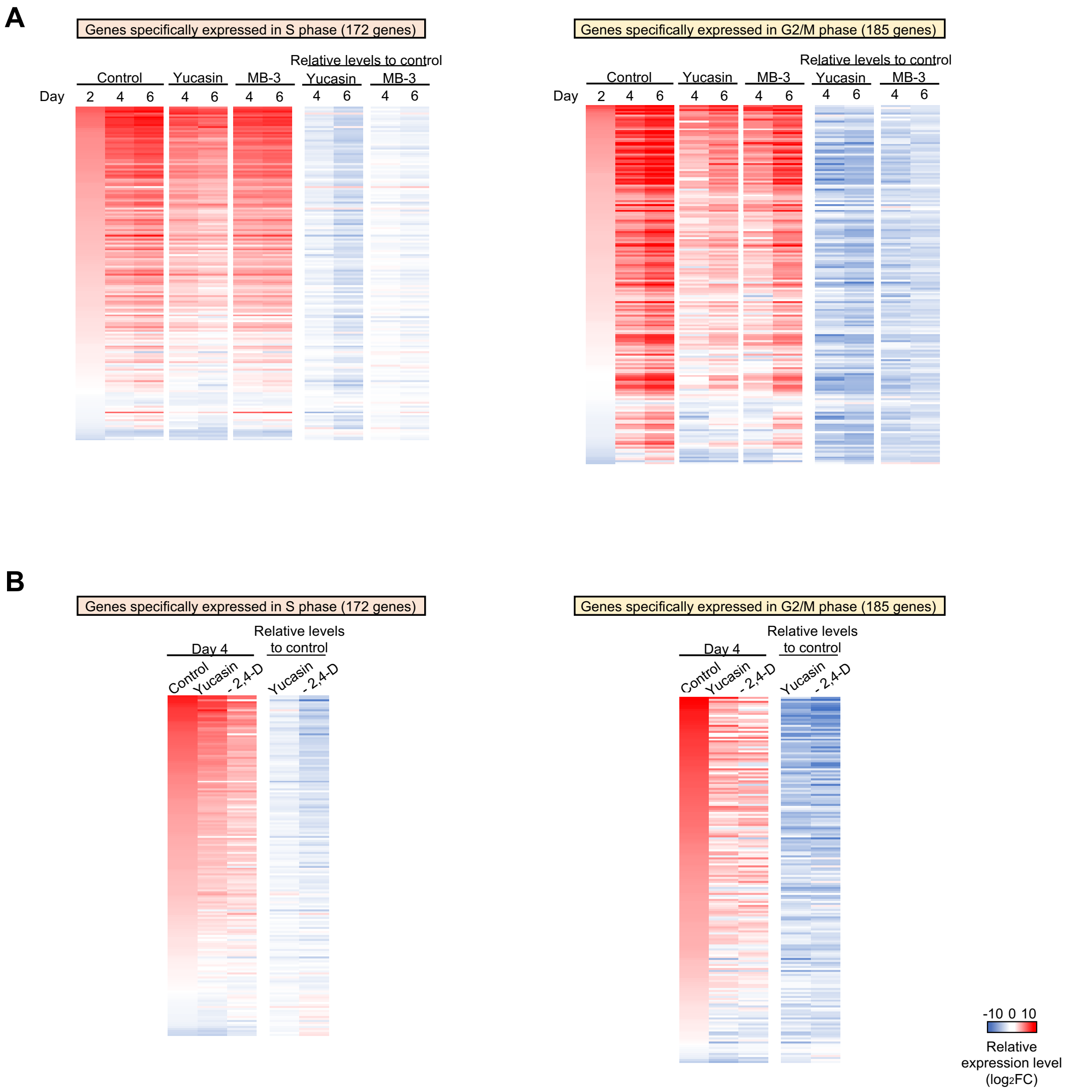


**Supplemental Figure S7.**

**Roles of histone acetylation, auxin biosynthesis and exogenous 2,4-D in regulating expression of cell cycle genes during protoplast reprograming.** (**A**) Heatmap representing the transcriptional changes for 172 genes specifically expressed at S phase and 185 genes specifically expressed at G2/M phase. The left three columns show expression levels in the control, 50 μM yucasin, and 50 μM MB-3 condition as values normalized ($log_2FC$) to Day 0. The ‘Relative levels to control’ columns show the normalized expression levels in the yucasin or MB-3 condition compared to the control condition for respective time points as relative values ($log_2FC$). Gene sets are taken from Kobayashi et al. (2015). Two genes in the original gene set were omitted from the heatmap of S phase genes since they were not expressed in all samples (see Supplemental Data Set S8B). (**B**) Heatmap representing the transcriptional changes for the same gene sets as in (A). The left three columns show expression levels at Day 4 in the control, 50 μM yucasin, and 2,4-D-omitted (named '- 2,4-D') condition as values normalized ($log_2FC$) to Day 0. The ‘Relative levels to control’ columns show the normalized expression levels in yucasin or - 2,4-D condition compared to the control condition for respective time points as relative values ($log_2FC$). Two genes in the original gene set were omitted from the heatmap of S phase genes since they were not expressed in all samples (see Supplemental Data Set S8D). **Supporting Figure 6.**

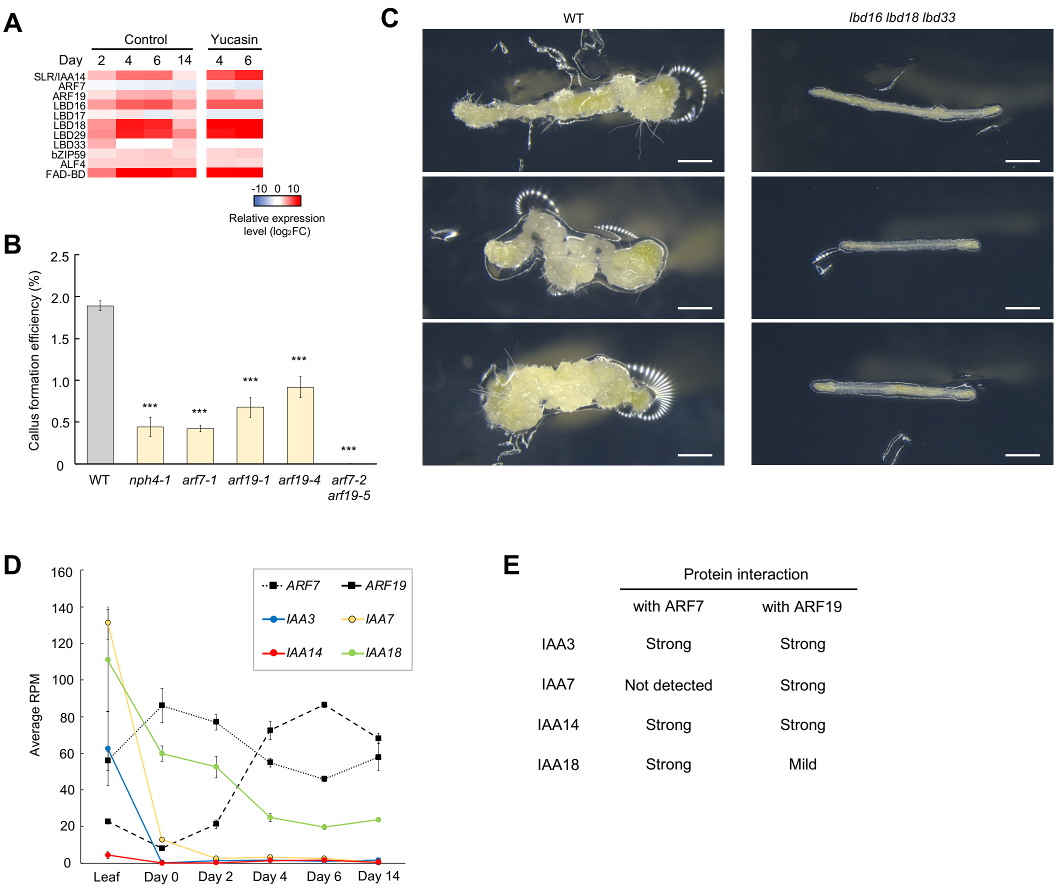


**Supplemental Figure S8.**

**Roles of auxin signaling components in protoplast cell cycle reinitiation.** (**A**) Heat map representing the transcriptional changes for genes implicated in auxin-induced callus formation in tissue culture. Expression levels at Day 2, 4 and 6 in the control condition are shown as values normalized ($log_2FC$) to Day 0. Gene sets are taken from Ikeuchi et al. (2019). (**B**) Callus formation efficiency of protoplasts isolated from WT, *arf7* and *arf19* mutants. Data are represented as mean ± SEM. n = 30 from 6 biological replicates for the WT and 15 from 3 biological replicates for the others. ***$P < 0.001$ (two-tailed Welch's *t*-test compared to WT). (**C**) Callus formation in hypocotyl explants from WT and *lbd16-1 lbd18-1 lbd33-1* seedlings incubated on CIM for 21 days. Callus formation was assessed using more than 80 explants from 2 biological replicates for each genotype and phenotypic reproducibility was confirmed. (**D**) Transcriptional changes for *Aux/IAA* genes. RNA-seq data are represented as average RPM ± SEM. n = 8 for Day 0 and n = 3 for others. (**E**) Protein interaction of Aux/IAA with ARF7 and/or ARF19 based on Piya et al. (2014). Scale bars are 1 mm (C). **Supporting Figure 7A-D.**

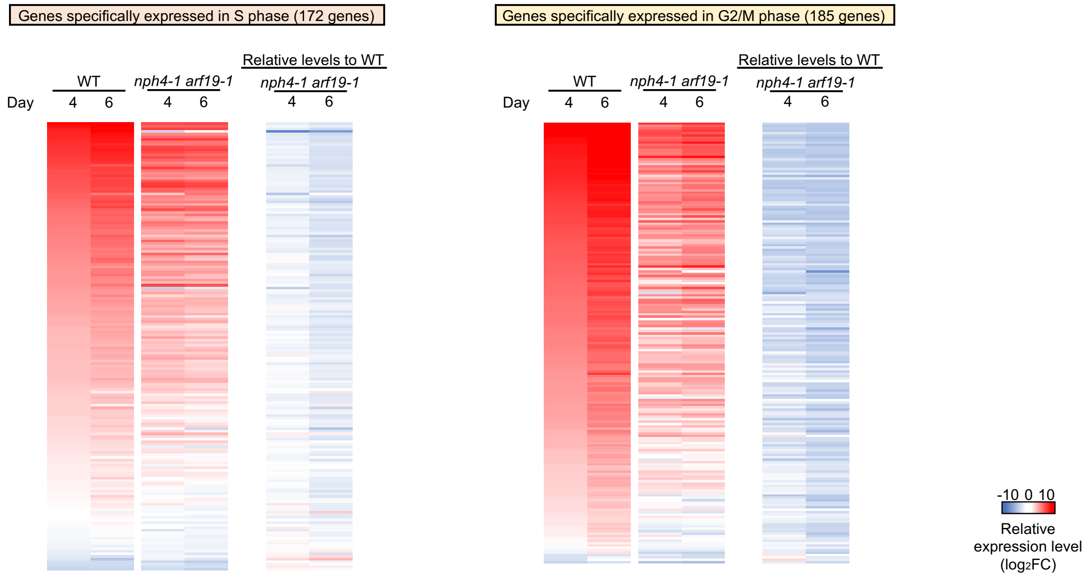


**Supplemental Figure S9.**

**Roles of ARF7 and ARF19 in regulating expression of cell cycle genes during protoplast reprograming.** Heatmap representing the transcriptional changes for 172 genes specifically expressed at S phase and 185 genes specifically expressed at G2/M phase. The left two columns show expression levels in the WT and *nph4-1 arf19-1* protoplasts as values normalized ($log_2$FC) to Day 0 of respective genotype. The ‘Relative levels to WT’ columns show the normalized expression levels in *nph4-1 arf19-1* compared to the WT for respective time points as relative values ($log_2$FC). Gene sets are taken from Kobayashi et al. (2015). Two genes in the original gene set were omitted from the heatmap of S phase genes since they were not expressed in all samples (see Supplemental Data Set S10B). **Supporting Figure 7E.**

## Supplemental Table S1.

## Composition of the media and reagents used in this study.

| Name | Compositions | pH | Solidifier | Sterilization | Storage | Phytohormones |
|---|---|---|---|---|---|---|
| GM | Half-strength Murasige-Skoog (MS) salts (Wako) supplemented with MS vitamins, 1 % (w/v) sucrose, 0.05 % (w/v) MES | 5.7 (by KOH) | 0.8 % (w/v) Gelzan CM-Gelrite (Sigma) | Autoclaving (121˚C, 20 min) | 4˚C | -- |
| Digestion Cocktail | 1 % (w/v) Cellulase Onozuka RS (Yakult), 0.1 % (w/v) Pectolyase (Kyowa or Sigma P3026*), 8 mM $CaCl_2 \cdot 2H_2O$, 0.4 M D(-)-mannitol | 5.5 (by HCl) | -- | Filtration (0.22 µm pore) | -20˚C | -- |
| Wash Medium 1 | 1 volume of 0.5 M D(-)-mannitol and 2 volumes of 0.2 M $CaCl_2 \cdot 2H_2O$ | -- | -- | Autoclaving (121˚C, 20 min) | Room temperature | -- |
| Wash Medium 2 | 2 volumes of 0.5 M D(-)-mannitol and 1 volume of 0.2 M $CaCl_2 \cdot 2H_2O$ | -- | -- | Autoclaving (121˚C, 20 min) | Room temperature | -- |
| Sodium Alginate Solution | 1.5 % (w/v) sodium alginate (300-400 cP, Wako), 0.4 M D(-)-mannitol | -- | -- | Autoclaving (121˚C, 20 min) | 4˚C | -- |
| $CaCl_2$ plate | 20 mM $CaCl_2 \cdot 2H_2O$, 0.4 M D(-)-mannitol | -- | 1 % (w/v) agar (Wako 010-15815) | Autoclaving (121˚C, 20 min) | 4˚C | -- |
| PCIM | Gamborg B5 salts (Wako) supplemented with B5 vitamins, 2 % (w/v) D(+)-glucose, 0.05 % (w/v) MES, 0.4 M D(-)-mannitol | 5.7 (by KOH) | -- | Autoclaving (121˚C, 20 min) | 4˚C | 1 mg/L 2,4-D, 176µg/L thidiazuron (added in time of use) |
| CGM | Gamborg B5 salts (Wako) supplemented with B5 vitamins, 2 % (w/v) D(+)-glucose, 0.05 % (w/v) MES, 0.4 M D(-)-mannitol | 5.7 (by KOH) | -- | Autoclaving (121˚C, 20 min) | 4˚C | 0.5 mg/L 2,4-D, 528µg/L thidiazuron (added in time of use) |
| SIM | Gamborg B5 salts (Wako) supplemented with B5 vitamins, 2 % (w/v) D(+)-glucose, 0.05 % (w/v) MES | 5.7 (by KOH) | 0.25 % (w/v) Gelzan CM-Gelrite (Sigma) | Autoclaving (121˚C, 20 min) | 4˚C | 0.15mg/L IAA, 0.5 mg/L 2-iP (added after autoclaving) |
| Citrate Solution | 20 mM trisodium citrate, 0.3 M D(-)-mannitol | -- | -- | Autoclaving (121˚C, 20 min) | Room temperature | -- |
| GM for Tissue Culture | MS salts (Wako) supplemented with 1 % (w/v) sucrose | 5.7 (by KOH) | 0.6 % (w/v) Gelzan CM-Gelrite (Sigma) | Autoclaving (121˚C, 20 min) | 4˚C | -- |
| CIM | Gamborg B5 salts (Wako) supplemented with B5 vitamins, 2 % (w/v) D(+)-glucose, 0.05 % (w/v) MES | 5.7 (by KOH) | 0.25 % (w/v) Gelzan CM-Gelrite (Sigma) | Autoclaving (121˚C, 20 min) | 4˚C | 0.5 mg/L 2,4-D, 0.1 mg/L Kin (added after autoclaving) |

*Both types of pectolyase yielded similar results at phenotypic level. For RNA seq analysis, only Kyowa's pectolyase was used.

## Supplemental Table S2.

## Primers used in this study.

| Primer names | Aims | Primer sequences (5' → 3') |
|---|---|---|
| attB4-pCAB3-Fw | *CAB3* promoter cloning | GGGGACAACTTTGTATAGAAAAGTTGAAATCAAGAGAAAATGTGATTC |
| pCAB3_attB1-Rv | *CAB3* promoter cloning | GGGGACTGCTTTTTTGTACAAACTTGTGAAACTTTTTGTGTTTTTTTTTTTT |
| attB4-pHAG1-Fw | *HAG1* promoter cloning | GGGGACAACTTTGTATAGAAAAGTTGAAGATTCGCCGAACTCAAGAAG |
| pHAG1_attB1-Rv | *HAG1* promoter cloning | GGGGACTGCTTTTTTGTACAAACTTGACGAAGCAGTATAGTGAAGGTG |
| TOPO-HAG1-CDS-Fw | *HAG1* CDS cloning | CACCATGGACTCTCACTCTTCCCACCT |
| HAG1-CDS-Rv | *HAG1* CDS cloning | TTGAGATTTAGCACCAGATTGG |
| TOPO-YUC1-CDS-Fw | *YUC1* CDS cloning | CACCATGGAGTCTCATCCTCACAA |
| YUC1-CDS-Rv | *YUC1* CDS cloning | TTAGGATTTAGAGGTAAAGAC |
| PP2A3-Fw | *PP2A3* RT-qPCR | TAACGTGGCCAAAATGATGC |
| PP2A3-Rv | *PP2A3* RT-qPCR | GTTCTCCACAACCGCTTGGT |
| YUC1-Fw | *YUC1* RT-qPCR | TTCCTAACGGCTGGAGAGGA |
| YUC1-Rv | *YUC1* RT-qPCR | TATTCCTGGTGGACCCCTTG |
| YUC4-Fw | *YUC4* RT-qPCR | TAACGAGGAACGGGGCAAAG |
| YUC4-Rv | *YUC4* RT-qPCR | GGCGTTTTTGGCATTCCTTCT |
| PLT3-Fw | *PLT3* RT-qPCR | CTACGATCCACGTCACCACC |
| PLT3-Rv | *PLT3* RT-qPCR | TCATCGACCTCTGAACCGGA |
| PLT5-Fw | *PLT5* RT-qPCR | GTTGTGTGGACAATATGAATGAT |
| PLT5-Rv | *PLT5* RT-qPCR | ATGACGAAAAACACCGTGGA |
