# Supplementary material for: Transcriptional activation of auxin biosynthesis drives developmental reprogramming of differentiated cells
Source: Plant Cell. 2022 Aug 4;34(11):4348–65. doi: 10.1093/plcell/koac218 (PMC9614439; doi:10.1093/plcell/koac218)
Supplement: koac218_Supplementary_Data [file tpc21.01024.PeerReviewReport.pdf › koac218_Supplementary_Data/tpc21.01024.PeerReviewReport.pdf]

Transcriptional activation of auxin biosynthesis drives developmental reprogramming of differentiated cells

Yuki Sakamoto, Ayako Kawamura, Takamasa Suzuki, Shoji Segami, Masayoshi Maeshima, Stefanie Polyn, Lieven De Veylder, Keiko Sugimoto

Corresponding author: Keiko Sugimoto (keiko.sugimoto@riken.jp)

Review timeline:

TPC2021-RA-00944	Submission received:	October 28, 2021
	1st Decision:	November 22, 2021 *manuscript declined (might reconsider)*
**TPC2021-RA-01024**	Submission received:	June 8, 2022
	1st Decision:	July 4, 2022 *accept with minor revisions*
**TPC2021-RA-01024R1**	1st Revision received:	July 6, 2022
	2nd Decision:	July 11, 2022 *acceptance pending, sent to science editor*
	Final acceptance:	July 18, 2022

REPORT: (The report shows the major requests for revision and author responses. Minor comments for revision and miscellaneous correspondence are not included. The original format may not be reflected in this compilation, but the reviewer comments and author responses are not edited, except to correct minor typographical or spelling errors that could be a source of ambiguity.)

TPC2021-RA-00537 1st Editorial decision – *declined* November 22, 2021

Thank you for choosing to send your manuscript entitled "Transcriptional activation of auxin biosynthesis drives developmental reprogramming of differentiated cells." for consideration at The Plant Cell. Your submission has been evaluated by members of the editorial board as well as expert reviewers in your field, and we regret to inform you that we are not able to recommend publication of this manuscript in its current form. We have not made this decision lightly. We have had input from multiple scientists, and we have solicited post-review comments as well. Our present policy is to offer streamlined decisions and to not advise on the direction of the work by requesting extensive modifications or substantial additional experiments.

However, we also felt that if you could address the major points raised by the reviewers by new experiments, we would welcome a resubmission. This may be treated as a new submission, but we would attempt to use at least some of the same reviewers. Nevertheless, reviewers will be asked to assess as a new manuscript (i.e. are the claims fully supported by the data and do the results presented move the field forward?), and not only whether previous reviewer comments have been addressed.

As you will read below, the reviewers raised a number of points that we felt would need to be addressed in a revision. In particular, Reviewer #2 has a number of auxin-centric considerations that we would like you to carefully address; some of these may require additional experiments whereas others can be easily resolved with changes to the text.

It will be important to convince the editors and reviewers that the study adds significant new understanding(s) of mechanisms or processes, or otherwise represents a significant advance in the field, and that the major claims made are fully justified by the data presented. This includes careful consideration and explanation of the various controls used in experiments, the extent and manner of replication, and the statistical analyses used. Sampling methods and the nature of "biological replicates" should be described precisely (i.e. different plants, parts of plants, pooled tissue, independent pools of tissue, sampled at different times, etc.), along with a clear description of and rationale for any statistical analyses conducted. The reader should know exactly what was sampled; what forms the basis of the calculation of any means and other statistical variables and parameters reported. This is also necessary to ensure that proper statistical analysis was conducted.

-- Reviewer comments:

Reviewer comments on previously declined manuscript and **author responses:**

Reviewer #1:

The findings by Sakamoto et al., reveal the role of local auxin biosynthesis in instructing the cell division in isolated protoplast, a fundamental step towards acquisition of pluripotency. The authors use a combination of biochemistry, cell biology and genetics to establish the role of the local hormonal environment in the protoplast regeneration and how local auxin produced in the protoplasts exert effects distinct from the auxin (synthetic auxin, 2,4D) applied externally in fine tuning the cell division. I enjoyed reading their nice work. The authors can consider the following points and revise their manuscript.

Point 1. While I like their logical speculation of a link between increase in auxin production and activation of ARF7/ARF19, I suggest they should provide some experimental evidence towards that. They can assess the transcript levels of ARF in response to XVE-YUC1 induction (optimal induction sufficient to increase in callus formation) and determine if there is a activation of ARF expression.

RESPONSE: We agree that this is an important point. Our RNA-seq data on yucasin-treated protoplasts indeed show that auxin production is required for the expression of *ARF7* and *ARF19* (Figure 7A and Supplemental Data Set 9A). We thus added the following sentences in the Results section.

"Additionally, we found that the expression of *ARF7* and *ARF19*, the activators of auxin signalling, is reduced by yucasin treatment and in contrast, the repressor *IAA14* level is increased (Figure 7A and Supplemental Data Set 9A), suggesting that they are involved in the downstream signalling pathways of IAA biosynthesis-mediated protoplast division."

Point 2. In light of recent studies by Xu et al, 2021 (Science Advances) where stochastic burst of expression of battery of genes upon protoplast isolation is key to protoplast regeneration, it is interesting to see that some of these genes such as PLT7 could contribute to increase in local auxin production. PLT7 is known to activate local auxin biosynthesis during leaf vascular regeneration. While studies by Xu et al., 2021 show the activation of a variety of key genes, the present study reveals the mechanisms by which these genes could act in growth of protoplast i.e., by activating auxin biosynthesis which in turn instructs the proper cell division. Authors can discuss this to give a holistic view.

RESPONSE: We thank the reviewer for this great suggestion. Our new data indeed show that PLT induction is regulated by histone acetylation and they consequently activate auxin biosynthesis to promote protoplast reprogramming (Figure 5). We added the following section in the Results section to describe these new data.

"Histone acetylation-mediated activation of PLTs is responsible for *YUC* upregulation.

We next investigated how histone acetylation regulates the YUC-mediated IAA biosynthesis. Given that *YUC* locus are not strongly acetylated in several regeneration contexts (Rymen et al., 2019; Kim et al., 2018), we searched for upstream transcriptional regulators that can be activated by histone acetylation. Among several transcriptional regulators that can induce *YUC* expression, we decided to focus on PLT3, PLT5 and PLT7, transcriptional activators of *YUC4* in wound-induced vascular regeneration (Radhakrishnan et al., 2020) since their locus are acetylated in the tissue culture condition (Kim et al., 2018) and their expression levels are sharply upregulated in cultured WT protoplasts compared to Day 0 (Figure 5A). We also found that among them, the expression of most strongly upregulated *PLT3* is compromised by both MB-3 treatment and *hag3-1* mutation (Figure 5A and 5B).

To explore an involvement of PLTs in protoplast reprogramming, we first examined whether PLTs are required for YUC-mediated callus formation. We observed that *plt3 plt5 plt7* mutants (Prasad et al., 2011) display reduced callus formation efficiency and importantly, this impairment can be partially rescued by ectopic *YUC* expression driven by *PLT5* promoter (Radhakrishnan et al., 2020) (Figure 5C). Consistently, RT-qPCR analysis showed that the upregulation of *YUC1* is less prominent in *plt3 plt5 plt7* protoplasts at Day 4 and Day 6 (Figure 5D), when *YUC1* expression is essential for the promotion of callus formation (Figure 3E). Unlike the case in vascular regeneration (Radhakrishnan et al., 2020), we did not detect significant alterations of *YUC4* expression by *plt* mutations (Figure 5D). We further observed that protoplasts isolated from *plt3 plt5 plt7* and *pPLT5::YUC4-YFP/plt3 plt5 plt7* plants are

less sensitive to MB-3 treatment compared to WT (Figure 5C). Notably, *pPLT5::YUC4-YFP/plt3 plt5 plt7* protoplasts showed better callus formation efficiency than *plt3 plt5 plt7* even in the presence of MB-3 (Figure 5C), supporting that the PLT-YUC activation is the core downstream pathway regulated by histone acetylation. Collectively, these results suggest that PLTs are transcriptionally upregulated through histone acetylation and they promote protoplast division by increasing the *YUC* expression."

We also added the following sentences in the Discussion section.

"We also demonstrate that there is key difference in how IAA biosynthesis is activated since our data suggests that PLTs increase IAA biosynthesis through transcriptional activation of *YUC1* (Figure 5), not *YUC4* as in vascular regeneration (Radhakrishnan et al., 2020). What differences, e.g., the cell types being reprogrammed and/or the physiological environments surrounding those cells, are reflected in such diverse pathways and functions, is an important question to be comprehensively answered in the future."

Point 3. For Figure 4 C, n= 9 for control and n= 7 for experimental sample. How many out of 7 or 9 protoplasts display the DR5-GFP expression pattern and typical cell division pattern as shown in the figure?

RESPONSE: 9 and 7 are the total numbers of microscopic fields (region of interests, ROIs) we observed in the 2 independent experiments. We observed 902 protoplasts from 9 ROIs for the control condition and 730 protoplasts from 7 ROIs for the yucasin condition (Figure S6B), which means that each ROI contained around 100 protoplasts. To show the average ratios of DR5-GFP-positive protoplasts, we calculated the percentages of DR5-GFP-positive protoplasts among the total protoplasts in each ROI and showed their average values in Figure 4D. We added the following sentence in the corresponding figure legend to clarify this point.

"Error bars represent standard error. n = 9 region of interests (ROIs) containing 902 cells in total for control and 7 ROIs containing 730 cells in total for yucasin-treated protoplasts from 2 independent experiments."

Reviewer #2:

This study investigated the process of de-differentiation of mesophyll protoplasts and callus formation in tissue culture conditions using transcriptomics and microscopy on a series of auxin and histone acetyltransferase (HAT) mutants, transgenic lines, and wild-type plants treated with auxin, auxin inhibitors and HAT inhibitors. Not surprisingly, the authors arrive to the conclusion that auxin biosynthesis (in addition to exogenous auxin provided in the media) and chromatin reprogramming are both very important for protoplast regeneration and efficient callus formation, the conclusions that are expected and therefore not very exciting. A lot of very painstaking, routine work is presented, such as cell shape measurements and the phenotypic classification of hundreds of cells into different cell types/fates based on marker gene expression and cell morphology. The work is carefully executed but is largely limited to the morphological and transcriptomic assessment of the regenerating protoplasts. Mechanistically, no novel functional relationships are uncovered, with previously established pathways important for cell reprogramming and division in other developmental contexts now implicated in protoplast regeneration and callus formation.

Point 1. Some of the conclusions drawn, such as the differential effects of exogenous 2,4D versus endogenously produced auxin on the expression of stage-specific cell cycle genes, appear to be preliminary and are not followed up in any way at the mechanistic level. The most unexpected and thus interesting result is the differential effects of stabilizing mutations in protoplast/callus-expressed IAA genes on callus formation, but the severity of the mutant defects is not correlated with the transcriptional regulation of these genes in wild-type plants and no explanations are provided. The final model presented in figure 7 is very helpful to systematize the findings of the paper, but also makes it obvious that this study is largely descriptive. Nonetheless, the work reported provides an important foundational framework for the molecular timeline of protoplast regeneration from the cell division perspective. Other key processes, such as the epigenetic reprogramming or the transcriptional changes orchestrating cell wall regeneration, are left completely unexplored.

RESPONSE: We thank the reviewer for this critical comment. To address this concern, we provide two new sets of data in the revised manuscript, one to show that histone acetylation is required to activate *PLT* expression, leading to the induction of downstream *YUC1* gene (Figure 5), and the other to show that IAA-induced ARF7 and ARF19 are required to activate *MYB3R4* expression (Figure 7). These functional relationships have not been described in previous studies, thus we believe this study now provides stronger mechanistic insights into how new auxin

production is regulated and how auxin in turn regulates the timely progression of cell cycle in development. We want to note that our next question is how direct these regulations are, for instance, whether histones are acetylated at *PLT* locus and whether ARFs directly regulate *MYB3R4* expression by binding to its promoter sequence. We believe addressing these questions is beyond the scope of this manuscript and we hope to perform chromatin immunoprecipitation experiments with small populations of reprogramming protoplasts in our future study.

Point 2. Line 80, define "high viability" by stateing the % of surviving cells.

RESPONSE: We provided this information in the legend for Supplemental Figure 1A but we see it was not easy to find. We thus added the following sentence in the Results section.

"Protoplasts were isolated from mature rosette leaves with high viability (98.66 ± 0.19 %), embedded into sodium alginate gels and cultured in protoplast callus induction medium (PCIM) supplemented with 2,4-D and thidiazuron as auxin and cytokinin, respectively (Figure 1A, Supplemental Figure 1A and Supplemental Table 1)."

Point 3. Figure S1, in panel E, I am confused by the flow chart starting with 10do and ending with 0do cells (should not the direction of arrows be switched?). How many of the 164 cells still show the eGFP marker at 10do? In panel H, what phenotypic characteristics were used to infer that the protoplast originates from a guard cell?

RESPONSE: Supplemental Figure 1E : We apologize for the confusion but in this experiment we first identified 164 cells that divided by Day 10 and then tracked back the same cells in the photographs we took at Day 0 to examine their differentiation status at Day 0. None of the 164 cells display eGFP signals at Day 10 since they no longer have the mesophyll identity. To clarify this point, we added the following sentences in the corresponding figure legend.

"Among 164 cells that divided by Day 10, 160 cells were eGFP positive and had mesophyll cell-like appearance at Day 0. Among the 4 cells that were eGFP negative at Day 0, 2 cells had mesophyll cell-like appearance and the other 2 had guard cell-like appearance based on the density of chloroplasts."

Supplemental Figure S1H: We used the density of chloroplasts to infer guard cells since protoplasts isolated from guard cells have less chloroplasts compared to mesophyll protoplasts (Pandey et al. New Phytol. 2002). We revised the corresponding sentence in the Results section as below.

"The two remaining H2A-eGFP-negative protoplasts also contain chloroplasts but they resemble guard cells based on their lower chloroplast density than those in mesophyll cells (Panday et al., 2002) (Supplemental Figure 1H)."

Point 4. Figure S2, in panel B, is the shrinking cell alive or dead? Do the shrinking cells have their cell walls regenerated and, if yes, how does the dynamics of this process differ between those cells that go on to grow and divide and those that shrink?

RESPONSE: We think the shrinking cells are probably dead since they express strong autofluorescence at later time points. Although we could not investigate cell wall regeneration in protoplasts embedded in the sodium alginate gels (as the gels impeded the penetration of fluorescent dyes such as Calcofluor White), a previous study showed that cell wall regeneration occurs within 2 days after protoplast isolation (Kuki et al., 2017). Since many cell wall biosynthesis genes are upregulated by Day 2 (Supplemental Data Set 2) and many protoplasts start to shrink by Day 2 (Supplemental Figure 3B), it is likely that the shrinking cells fail to regenerate cell walls and thus have much weaker mechanical strength. To address this issue, we revised the corresponding sentences in the Results section as below.

"A previous study reported that cell wall regeneration occurs within 2 days after protoplast isolation (Kuki et al., 2017). Consistently, our RNA-seq data showed that many cell wall biosynthesis genes, including *CELLULOSE SYNTHASE*s (*CESA*s) and *CELLULOSE SYNTHASE-LIKE*s (*CSL*s), are sharply upregulated by Day 2 (Supplemental Data Set 2). Since many protoplasts start to shrink by Day 2 (Supplemental Figure 3B), cell wall regeneration might be a key step for protoplasts to reacquire the mechanical strength at early steps and advance the subsequent reprogramming processes."

Point 5. Lines 132-133, how can the conclusion that the reprogramming starts "during protoplast isolation" be made based on the transcriptional profiles of 0-day protoplasts relative to intact leaves given that the protoplast cell population is largely devoid of epidermal and vascular cells that contribute to the gene expression profile of intact leaves?

RESPONSE: We agree that we cannot make quantitative assessments between these datasets since intact leaf samples contain other cell types. We want to emphasize, however, that most of epidermis and vascular cells do not contain chloroplasts thus if the expression of chloroplast genes remains unchanged during protoplast isolation, their transcript levels should increase in mesophyll-enriched samples. As shown in Supplemental Figure 2, we found the opposite, downregulation of chloroplast genes in freshly isolated protoplasts, thus qualitatively suggesting that cellular dedifferentiation already starts during protoplast isolation. To clarify this issue, we revised the following sentences in the Results section.

"Since leaf samples contain other cell types such as epidermis and vascular cells that do not contain chloroplasts, we cannot make quantitative assessments between these datasets. It was, however, clear that many genes encoding photosynthetic components, including subunits of light harvesting complexes, are downregulated during protoplast isolation and/or following several days of incubation."

Point 6. Figure 2, in panels A and B (and Figure S4A), what is the specificity of MB-3 and was the general toxicity of this chemical ruled out (given that any mildly toxic chemical is expected to reduce or abolish callus formation much like MB-3 did)? Were the MB-3 cells shown in panel B tested for cell viability? The same concern applies to C646 and garcinol treatments in figure S4B.

RESPONSE: A previous study reported that MB-3 inhibits the GNAT/MYST family histone acetyltransferases in Arabidopsis (Aquea et al., 2017). We have also shown that MB-3 targets specific sets of genes without changing overall histone acetylation status (Rymen et al., 2019). In addition, we have unpublished data that show MB-3 promotes callus formation from Arabidopsis root explants, much like the increased callus formation reported for *hag1* mutants (Kim et al., 2018), strongly suggesting that MB-3 is not generally toxic to plant cells. As suggested by the reviewer, we also tested the viability of MB-3-treated cells by FDA staining and confirmed that the viability is comparable between control and MB-3 condition (Supplemental Figure 4G). We also examined the expression of genes involved in primary metabolism, which should reflect cellular viability, and confirmed that they are also comparable between the two conditions (Supplemental Data Set 5). These data together support that MB-3 does not have general toxic effects on the protoplasts. We described these data in the Results section as follows.

"We detected more than 7,000 DEGs for both control and MB-3 conditions compared to the freshly isolated protoplasts (Figure 2C, Supplemental Figure 4F and Supplemental Data Set 4). Importantly, the number of downregulated genes is comparable between control and MB-3 conditions (Supplemental Figure 4F). In addition, the expression level of key metabolic genes, for instance those encoding components of TCA cycle and respiratory chain, that are linked with cellular survival (Martínez-Reyes and Chandel, 2020) is overall similar in the control and MB-3 condition (Supplemental Data Set 5), implying that the cellular physiological status of MB3-treated protoplasts is not severely compromised. Consistently, the FDA staining showed the comparable viability of the protoplasts cultured in the two conditions (Supplemental Figure 4G), further supporting that MB-3 does not have general toxic effects on the protoplasts."

Similarly, we have previously shown that C646 does not inhibit callus formation from wounded hypocotyls (Rymen et al., 2019), thus we assume it is not toxic to plant cells. We added the following sentence in the Results section to state that the target specificity of C646 and garcinol is not known in plants.

"Although the target specificity of these inhibitors is not well worked out in plants, these data imply that multiple HATs from different subfamilies contribute to protoplast reprogramming."

Point 7. Lines 150-154, given the key roles of specific HAT genes in callus formation, it would be helpful to correlate the severity of regeneration defects in the mutants with the levels/patterns of HAT gene expression in regenerating WT protoplasts.

RESPONSE: As suggested by the reviewer, we examined the expression of HAT genes in regenerating WT protoplasts and found that there is no close correlation between their expression levels and the severity of mutant phenotypes (Figure 2A, Supplemental Figure 4C-D and Supplemental Data Set 3). We thus think that these HAT activities are regulated at the levels other than transcription. We added the following sentences in the Results section to describe these data.

"Interestingly, we do not see any clear correlation between these single HAT mutant phenotypes and the expression patterns of HAT genes in the control culture condition (Figure 2A, Supplemental Figure 4C-D and Supplemental Data Set 3). It is thus likely that these HAT activities are regulated at the levels other than transcription."

Point 8. Line 158, given the celluar heterogeneity, this would have been a good case to do single-cell RNA-seq (this is just a thought, I am not expecting you to have that data).

RESPONSE: We agree that single-cell RNA-seq analysis is needed for our future work to further clarify the transcriptional differences between dividing and non-dividing cells. We thus added the following sentence in the Discussion section.

"Single-cell RNA-seq analysis will also be a great tool to investigate transcriptional differences between dividing and non-dividing cells."

Point 9. Line 161, it would be helpful to state how many genes are downregulated by MB-3 treatment (as a proxy for MB-3 toxicity and the downstream effects of chromatin relaxation allegedly triggered by this chemical).

RESPONSE: The number of downregulated genes is comparable between control and MB-3 conditions (2,719 genes in the control and 2,953 genes in the MB-3 condition, Supplemental Figure 4F), implying that MB3 affects some specific pathways rather than having general toxic effects. We added the following sentence in the Results section to describe these data.

"Importantly, the number of downregulated genes is comparable between control and MB-3 conditions (Supplemental Figure 4F)."

Point 10. Figure 3A, given the possibility of general toxicity of MB-3 being the reason for gene expression downregulation, it would be very helpful to confirm the selective transcriptional inhibition of auxin biosynthesis genes in HAT mutants by RT-qPCR. Auxin reporter analysis would also have been helpful in these mutant backgrounds.

RESPONSE: As suggested by the reviewer, we provide new RT-qPCR data that show downregulation of the *YUC1* gene in *hag3-1* protoplasts (Supplemental Figure 4H). Although we did not perform auxin reporter analysis in HAT mutants, we think we provide sufficient evidence to show that MB-3 is not generally toxic to plant cells and one key pathway affected by MB-3 is YUC-mediated auxin biosynthesis. We added the following sentence in the Results section to describe these data.

"Consistently, RT-qPCR analysis on cultured *hag3-1* showed a similar reduction of *YUC1* transcripts (Supplemental Figure 4H), supporting that HATs regulate the transcriptional activation of auxin biosynthesis in protoplast reprogramming."

Point 11. Lines 173-174, the results are as expected (I would have been very surprised if this was not found to be the case). The question is whether extra auxin supplied exogenously (beyond the standard level of 2,4D in the callus induction media) can revert the inhibitor-triggered regeneration defects.

RESPONSE: As suggested by the reviewer, we provide new data showing that exogenous IAA application partially rescues the regeneration defects caused by yucasin treatment (Supplemental Figure 5B). We added the following sentence in the Results section to describe these data.

"Importantly, the inhibitory effect of yucasin was partially rescued by exogenous IAA application (Supplemental Figure 5B), suggesting that optimum IAA level is required to induce reprogramming."

Point 12. Lines 175-180, I do not understand the problem with working on a segregating population of yuc1246 plants. The quads can easily be identified phenotypically (smaller rosettes, with curly leaves) in soil (Cheng et al, 2006). If the phenotypic differences are not obvious in tissue culture, plant genotyping of T-DNA mutants is straightforward, and the leaves of the desired mutant combinations can then be used for mesophyll protoplast isolation and callus formation. What am I missing here? Also, given that YUC1 appears to be the predominantly expressed YUC gene in these cell cultures, it would have made sense to examine the behavior of a single yuc1 mutant that is perfectly fertile, or double and triple (e.g. yuc124) mutant combinations containing YUC1 reported by Cheng et al, 2006. I would think that taa1 tar2 double and taa1 single auxin-deficient mutant would also display a severe regeneration defect (despite these two genes not showing strong repression by MB-3), as proper auxin levels need to be maintained. Kynurenine treatment data are consistent with that assumption.

RESPONSE: We agree that it would have been ideal to include data on *yuc1246* mutants but we could not identify

them phenotypically in our culture condition. We need to PCR genotype more than 200 seedlings (to identify >50 homozygous plants) for one protoplasting experiment but we could not perform this aseptically and keep the protoplast culture for 14 days. We did examine the phenotypes of *yuc1* and *yuc4* single mutants but neither of them showed the phenotype (Supplemental Figure 5C) likely because other YUCs can compensate for the loss of single *YUC* gene. We do, however, provide additional data to show that YUC-mediated IAA biosynthesis is one key pathway regulated by histone acetylation (Figure 5C) and increasing the IAA level is central to initiate protoplast reprogramming (Supplemental Figure 5B). Therefore, while our data do not demonstrate the specific requirement of YUC1 in protoplast reprogramming, they do uncover an important regulatory mechanism to optimize YUC-dependent IAA production in protoplasts. We revised the following sentence in the Results section to describe data on the *yuc* single mutants.

"As expected, multiple mutation of *YUC* genes using *yucQ* (*yuc3 yuc5 yuc7 yuc8 yuc9*) mutants (Chen et al., 2014) showed reduced callus formation efficiency (Figure 3C), although the single *yuc* mutants we tested did not show the phenotypes (Supplemental Figure 5C). "

Point 13. In that regard, in Figure 3C,D, can external addition of auxin beyond the concentration of 2,4D contained in the callus induction media complement the auxin deficiency of yuc and cyp79 mutants? Based on Figure S4G, exogenous auxin can improve callus regeneration efficiency in wild-type plants.

RESPONSE: As we discussed above, exogenous IAA application partially rescues the regeneration defects caused by yucasin treatment (Supplemental Figure 5B). Although we did not perform the same rescue experiments for *yuc* and *cyp79* mutants, we think we now provide enough data to show that optimum IAA level is required to induce reprogramming. We added the following sentence in the Results section to describe the yucasin-IAA data.

"Importantly, the inhibitory effect of yucasin was partially rescued by exogenous IAA application (Supplemental Figure 5B), suggesting that optimum IAA level is required to induce reprogramming.

Point 14. Line 188-193 and Figure 3E, regarding the authors' interpretation of higher level of YUC1 not being optimal for callus regeneration, it seem that the less dramatic effect of 5uM ED relative to 0.1uM ED may in part be the consequence of ED (or solvent) toxicity, as evidenced by the reduced efficiency of callus formation at the highest dose of ED in WT plants, especially treated with ED on Day 0 (even if the differences in the values are not statistically significant). In that regard, what concentration of DMSO was used for the control samples - is the solvent-only control equivalent to the amount of DMSO in 0.1uM ED or 5uM ED? This needs to be clearly stated in the figure legend.

RESPONSE: The concentration of DMSO is identical in all experiments since we always add 4 µL of DMSO, containing different levels of ED, to the 4 mL of PCIM, thus very likely that there is no solvent toxicity. We agree that 5uM ED may have some toxic effects but as pointed out by the reviewer, it is not statistically significant and we think that it does not affect our conclusion that the timing and dose of *YUC1* expression is important. We also want to add that our IAA feeding experiments also support this conclusion since adding IAA beyond the optimal range starts to inhibit callus regeneration (Supplemental Figure 5D). We added this information to the corresponding figure legend as below.

"4 µL of DMSO with or without β-estradiol (ED) was added to PCIM at the indicated final concentration at Day 0 or Day 4 of culture."

Point 15. Figure S4G, what is the IAA solvent (EtOH) concentration? Can the authors rule out EtOH toxicity at higher IAA concentrations?

RESPONSE: Yes, it is very unlikely that there is solvent toxicity because we always add 4 µL of EtOH, containing different levels of IAA, to the 4 mL of PCIM. We added this information to the corresponding figure legend as below.

"4 µL of EtOH with or without IAA was added to PCIM at the indicated final concentration at Day 0."

Point 16. Figure S4H, state the concentration of DMSO in the control sample and how it relates to the DMSO concentration in ED-treated samples.

RESPONSE: We add 3 µL of DMSO, containing different levels of ED, to the 3 mL of 1/2MS medium. We added this information to the corresponding figure legend as below.

"WT and *XVE-YUC1* seeds were sown in 3 mL of liquid half-strength MS medium and incubated at 22°C under light with rotation on a Shake-LR (TAITEC) for 10 days. 3 µL of DMSO with or without ED was added to the MS medium at the indicated final concentration and plants were grown for another 24 hours."

Point 17. Figure 4A and lines 200-202, how do YUC genes behave at later time points and why only the early time points shown if the authors have the RNA-seq data 14 (as shown in figure 3A plots)?

RESPONSE: As shown in Figure 3A, the expression of *YUC* genes remains high at Day 14. We revised the corresponding sentence as below to include this information.

"Indeed, our RNA-seq data show that IAA biosynthesis genes start to be upregulated before initial cell division and the high expression levels last for Day 14 (Figure 3A, 4A and Supplemental Data Set 7).

Point 18. Lines 205-206 and Figure 4B, this statement is misleading, as yucasin inhibits callus formation when added at any stage shown, and the definition of "strong" inhibition is arbitrary. It will be more accurate to state that yucasin has a stronger effect when added prior to the initial cell division.

RESPONSE: We agree that yucasin also has inhibitory effects for successive cell division. We revised the corresponding sentences in the Results section as follows.

"We therefore hypothesized that IAA biosynthesis promotes initial cell division. Notably, yucasin inhibits callus formation more strongly when it is added by Day 4, i.e. before most cells reinitiate the cell cycle, than when it is added later on (Figure 4B). This indicates that endogenous IAA production is essential for initial cell division, although it is also required for successive divisions."

Point 19. Figure 5C, please label the genes as involved in the S versus G1/S-phase (as in Figure 5A) and separate them into blocks for easy reference. What is the yucasin concentration used? This should be clearly stated.

RESPONSE: As suggested by the reviewer, we labelled the genes in Figure 5C. We used 50 µM yucasin and we added this information in the Results section.

Point 20. Lines 259-260 and 268-270, this proposition does not make sense to me as phrased. I do not think the differences between 2,4D and native auxin are necessarily due to their different sources (exogenous versus endogenous, as 2,4D presumably acts by first being taken up and perceived by the cell internally), but rather due to the differences in these auxins' chemical structures and accordingly, degradation dynamics, transport, receptor binding properties, etc.

RESPONSE: We agree that the chemical and/or physiological properties of 2,4-D and IAA might contribute to their different requirements we observed. We revised the corresponding sentences in the Results and Discussion sections as below.

"Previous studies have indicated that exogenous auxin regulates the G1/S transition at both transcriptional and post-translational levels in protoplasts (Carle et al., 1998; Pasternak et al., 2000), raising the possibility that exogenously supplied 2,4-D and endogenously produced IAA regulate different phases of the cell cycle to reinitiate cell division in protoplasts."

"These results therefore suggest that 2,4-D and IAA may play distinct roles during the initial division of protoplasts, where they transcriptionally activate the S phase and G2/M phase progression, respectively."

"Previous studies suggest that 2,4-D and IAA are transported differently through plasma membranes and tonoplasts (Delbarre et al., 1996; Ranocha et al., 2013) and they are also metabolized differently (Eyer et al., 2016), implying that some of these biochemical properties have previously uncharacterized physiological implications that can activate a specific auxin response pathway. Our data are also consistent with the idea that transcriptional activation of G2/M genes requires higher levels of auxin than those required for the G1/S gene activation. In this scenario, exogenously supplied 2,4-D might be sufficient to drive the G1/S transition but both 2,4-D and endogenously produced IAA are required for the G2/M transition."

Point 21. Lines 260-270, I am not convinced that the two treatments, yucasin versus no-2,4D, are directly comparable. The milder effects of yucasin may simply mean higher levels and activity of auxin in the cell (due to the imported 2,4D) relative to the no-2,4D treatment where only endogenously made auxins are available. And different levels (and the nature) of auxin in the cell may well have different effects of auxin-regulated gene expression.

RESPONSE: We agree that the transcriptional differences we observed in these two conditions may reflect gene expression under relatively high (yucasin) and low (no 2,4-D) auxin conditions. It is still very interesting that under yucasin conditions, G2/M genes are more strongly repressed than G1/S genes, implying that in this case, higher auxin levels are needed to transcriptionally activate G2/M genes. We discussed this possibility in the Discussion section as follows.

"Our data are also consistent with the idea that transcriptional activation of G2/M genes requires higher levels of auxin than those required for the G1/S gene activation. In this scenario, exogenously supplied 2,4-D might be sufficient to drive the G1/S transition but both 2,4-D and endogenously produced IAA are required for the G2/M transition."

Point 22. Figure 6D and S7, there seems to be no obvious correlation between the gene expression profiles of Aux/IAAs 3, 7, 14 and 18 and their role in callus formation. This needs to be explained. Transcriptional reporters may be helpful here.

RESPONSE: We apologize that the heatmap data were difficult to interpret. We now display RPM data to show that IAA3, 7 and 18 have strong expression in leaves and are downregulated during protoplasting and culture (Supplemental Figure 8D). Why only IAA3 and IAA18 mutations cause the callus formation defects is not clear but one key difference might be that according to Piya et al. (2014), IAA3 and IAA18 interact with both ARF7 and ARF19 while IAA7 interacts only with ARF19, thus allowing ARF7 to still function in *axr2-1* mutants. We included these interaction data (Supplemental Figure 8E) and revised the following sentences in the Results section.

" The expression of IAA7 is also strongly downregulated during protoplasting but its interaction is detected only with ARF19 and not with ARF7 (Piya et al., 2014) (Supplemental Figure 8E). The gain-of-function mutants for *IAA7*, *auxin resistant 2-1* (*axr2-1*) (Nagpal et al., 2000) does not cause obvious defects in callus formation (Figure 7D), suggesting that it does not play major roles in protoplast reprogramming."

Point 23. Figure S7C. The heat maps and RPM value changes appear to be inconsistent for some of the genes which could be due to the heat maps being plotted on the log 2 scale and the RPMs being plotted on a linear scale. For example, IAA18 appears to show no changes in gene expression between intact leaf, Day 0 and Day 2 protoplasts based on the white color of the heatmap blocks in Figure S7C, but the RPM values for this gene are shown to change from high in intact leaf to medium in Day 2 protoplasts. Likewise, IAA7 is mildly repressed both by protoplasting at Day 0 and further culturing at Day 2, but the RPM values in intact leaf versus Day 2 change from maximum red value to minimum green value (suggesting orders of magnitude difference?). Thus, the data representation appears to be unintentionally misleading (the same concern applies to other figures that combine heat maps in the log 2 scale and linear RPM values). It is also confusing to have Day 0 plotted against intact leaf samples but subsequent days of protoplasts grown in culture plotted against Day 0 in the heat maps. For example, in figure S7C, IAA10 and IAA16 appear to be induced by protoplasting at Day 0, but that they are repressed relative to Day 0 at later time points and it is difficult to infer whether the repression at Day 2, 4 etc. is comparable in strength to the initial induction at Day 0 unless all samples are normalized against the same "intact leaf" baseline. Why are the RPM values not plotted for all of the samples and are shown only for intact leaf and Day 2 protoplasts? I feel that plotting the actual RMP values for all samples rather than relative ratios may be more informative.

RESPONSE: We are sorry for this confusion. To address this issue, we now display only RPM data for IAA3, 7, 14 and 18 as dot plots (Supplemental Figure 8D). For Supplemental Figure 2, we show only heat maps plotted on the log 2 scale. For Supplemental Figure 2, we do want to keep two separate columns, one to display the transcriptional changes during protoplasting (Day 0/Leaf column) and the other to display the transcriptional changes during subsequent protoplast culture (Cultured protoplasts/Day 0). We revised the corresponding figure legend as below to clarify our intention.

"The leftmost column shows relative expression levels (log_2FC) in protoplasts at Day 0 compared to 23 DAS leaves to visualize the transcriptional changes during protoplast isolation. The second column shows relative expression

levels (log_2FC) in cultured protoplasts at Day 2, 4, 6 and 14 compared to Day 0 to visualize the transcriptional changes during subsequent protoplast culture."

Point 24. In other RNA-seq heat maps (e.g., in figure S5), it is not very helpful to plot "average RPM" values given that most genes are strongly differentially regulated.

RESPONSE: Following this comment from the reviewer, we removed average RPM values from heat maps.

Point 25. Lines 307-308, I do not think that can be concluded, as even later yucasin treatments (up to 6 days after protoplast isolation) have a clear effect on callus formation. Does additional callus continue to form from cells incubated past 14 days (the dynamics of callus formation should probably be plotted early on in the paper)? If yes, would it make sense to monitor yucasin-treated and untreated control cells past the standard 14 days?

RESPONSE: We agree, thus we toned down our statement as below. Our data show that the majority of protoplasts undergo the first cell division by Day 9 (Supplemental Figure 1J), thus we think monitoring cell division beyond Day 14 will not change our conclusion.

"Notably, auxin biosynthesis is essential for their first cell division while it is less critical for successive divisions (Figure 4B)."

Point 26. Lines 351-353, I do not think the data in support of this conclusion are very strong.

RESPONSE: We agree, thus we toned down our statement and discussed an alternative possibility in the Discussion section.

"Interestingly, our data suggest that 2,4-D and endogenously produced IAA may activate distinct phases of the cell cycle to reinitiate protoplast division (Figure 6C, Supplemental Figure 8B and Supplemental Data Set 8D-E)."

"Our data are also consistent with the idea that transcriptional activation of G2/M genes requires higher levels of auxin than those required for the G1/S gene activation. In this scenario, exogenously supplied 2,4-D might be sufficient to drive the G1/S transition but both 2,4-D and endogenously produced IAA are required for the G2/M transition."

Point 27. Lines 377-379, again, as stated above, intact leaves (with epidermis and vasculature in addition to mesophyll cells) are not directly comparable in terms of their transcriptional profiles to protoplasts predominantly represented by mesophyll cells.

RESPONSE: As we discussed above, we clarified this issue by revising the following sentences in the Results section.

"Since leaf samples contain other cell types such as epidermis and vascular cells that do not contain chloroplasts, we cannot make quantitative assessments between these datasets. It was, however, clear that many genes encoding photosynthetic components, including subunits of light harvesting complexes, are downregulated during protoplast isolation and/or following several days of incubation."

Point 28. Why was the cell wall regeneration process not examined in this study? When do the cell wall biosynthesis genes come up during the regeneration process and at what point do the cell walls reform? Do the shrinking cells that fail to reprogram and divide fail to make cell walls? In the future, single-cell RNA-seq can be employed to differentiate the transcriptional programs of protoplasts adopting different fates.

RESPONSE: We could not investigate cell wall regeneration in protoplasts embedded in the sodium alginate gels as the gels impeded the penetration of fluorescent dyes such as Calcofluor White. We also could not omit the gel embedding step since it was vital for protoplast reprogramming. As we discussed above, however, we have some evidence to support that the shrinking cells probably fail to regenerate cell walls and cannot not reacquire the mechanical strength to advance the subsequent reprogramming processes. We discussed this possibility in the Results section as below. We agree that single-cell RNA-seq will be ideal to transcriptionally differentiate protoplasts adopting different fates and we are planning to perform this experiment in our future study.

"A previous study reported that cell wall regeneration occurs within 2 days after protoplast isolation (Kuki et al., 2017). Consistently, our RNA-seq data showed that many cell wall biosynthesis genes, including *CELLULOSE SYNTHASE*s (*CESA*s) and *CELLULOSE SYNTHASE-LIKE*s (*CSL*s), are sharply upregulated by Day 2 (Supplemental

Data Set 2). Since many protoplasts start to shrink by Day 2 (Supplemental Figure 3B), cell wall regeneration might be a key step for protoplasts to reacquire the mechanical strength at early steps and advance the subsequent reprogramming processes."

TPC2021-RA-01024 1st Editorial decision – *revision requested* **July 4, 2022**

We have received reviews of your manuscript entitled "Transcriptional activation of auxin biosynthesis drives developmental reprogramming of differentiated cells.." On the basis of the advice received, the board of reviewing editors would like to accept your manuscript for publication in The Plant Cell. This acceptance is contingent on revision based on the comments of our reviewers. In particular, please address the minor typos, word changes, and figure improvements suggested by the reviewers.

-- Reviewer comments:

TPC2021-RA-01024R1 1st Revision received **July 6, 2022**

Reviewer comments on previous submission and **author responses**:

Reviewer #1

The authors have revised the manuscript substantially. They address all my comments adequately. The revised manuscript provides a transcriptional framework of local auxin biosynthesis. Interestingly, PLETHORA genes mediated transcriptional regulation can trigger the cell division in differentiated cells to generate pluripotent callus. It is tempting to speculate that ARF genes can act upstream of PLT genes in this transcriptional framework during cellular reprogramming. As a reader I will be happy to reads this manuscript in the Plant Cell.

Point 1. Fig. 4B - while the figure legend mentions statistical analyses done, no indicative symbol or letter has been provided to indicate the same in the figure graph.

RESPONSE: We did add asterisks to indicate the statistical significance in the graph shown in the right panel of Fig. 4B. The diagram in the left panel shows the timing of yucasin treatment, thus there is not statistics involved. To clarify this point, we revised the figure legend as below.

"Left diagram shows the timing of yucasin treatment."

Point 2. Fig. 2B - authors should specify if an equal volume of vehicle control (DMSO) has been added in control phenotype in the figure legend.

RESPONSE: We added the equal volume of DMSO with or without MB-3 to the cultures. We added this information to the figure legend as below.

Figure legend 2B: "4 µL of DMSO with or without MB-3 were added to PCIM at the indicated final concentration at the beginning of culture (Day 0)."

Point 3. Fig. 3B, 4D, 6B, 7B, 7C, 7D - for better clarity, authors should stick to using one type of statistical test for these plots. Given that in these figures there is variation in sample sizes between control and test groups, the Welch's t-test is appropriate and sufficient for suitable statistical interpretation of data.

RESPONSE: Following the reviewer's suggestion, we used the Welch's t-test for these data as well as some other data in Supplemental Figures. We revised the statistical symbols and figure legends accordingly.

Reviewer #2:

I reviewed an earlier version of this manuscript in 2021 (I was Reviewer 2). Back then, I left a number of comments and made several revision suggestions, some of which required additional experimental work. I was pleasantly surprised that the authors attempted to address nearly all of my criticisms, including several through new experimentation and figure (or figure legend) edits. With these revisions, the overall message stayed the same, but the clarity of data presentation and interpretation was much improved. I still feel that the study is painstakingly

routine, and the conclusions are mostly predictable and therefore not super-exciting. Nonetheless, the work was carefully executed and provides a solid framework for the events that take place during mesophyll protoplast reprogramming and cell regeneration into callus. Therefore, I believe the manuscript should be published in its current form (once the minor mistakes indicated below are fixed) and will provide an important reference point for the community on the molecular and phenotypic events that take place during mesophyll protoplast de-differentiation. I do not have any major new edits and most of my original criticisms have been addressed to my satisfaction.

TPC2021-RA-01024R1 2nd Editorial decision – *acceptance pending* **July 11, 2022**

We are pleased to inform you that your paper entitled "Transcriptional activation of auxin biosynthesis drives developmental reprogramming of differentiated cells." has been accepted for publication in The Plant Cell, pending a final minor editorial review by journal staff. At this stage, your manuscript will be evaluated by a Science Editor with respect to its presentation of scientific content, compliance with journal policies, and presentation for a broad readership.

Final acceptance from Science Editor **July 18, 2022**
